# Synthesis, Physicochemical Characteristics and Plausible Mechanism of Action of an Immunosuppressive Isoxazolo[5,4-e]-1,2,4-Triazepine Derivative (RM33)

**DOI:** 10.3390/ph14050468

**Published:** 2021-05-15

**Authors:** Marcin Mączyński, Andrzej Regiec, Aleksandra Sochacka-Ćwikła, Iwona Kochanowska, Maja Kocięba, Ewa Zaczyńska, Jolanta Artym, Wojciech Kałas, Michał Zimecki

**Affiliations:** 1 Department of Organic Chemistry, Faculty of Pharmacy, Wroclaw Medical University, 211A Borowska Street, 50-556 Wroclaw, Poland; andrzej.regiec@umed.wroc.pl (A.R.); aleksandra.sochacka-cwikla@umed.wroc.pl (A.S.-Ć.); 2 Laboratory of Immunobiology, Hirszfeld Institute of Immunology and Experimental Therapy, Polish Academy of Sciences, 12 Weigla Street, 53-114 Wroclaw, Poland; iwona.kochanowska@hirszfeld.pl (I.K.); maja.kocieba@hirszfeld.pl (M.K.); ewa.zaczynska@hirszfeld.pl (E.Z.); jolanta.artym@hirszfeld.pl (J.A.); wojciech.kalas@hirszfeld.pl (W.K.); michal.zimecki@hirszfeld.pl (M.Z.)

**Keywords:** isoxazole, isoxazolo[5,4-e]-1,2,4-triazepine, *N*-deuterated isotopologue, spectral analysis, structure research, TNFα, cell signaling, cyclooxygenase 2

## Abstract

Previous studies demonstrated strong anti-inflammatory properties of isoxazolo[5,4-e]-1,2,4-triazepine (RM33) in vivo. The aim of this investigation was to describe synthesis, determine physicochemical characteristics, evaluate biological activities in murine and human in vitro models, as well as to propose mechanism of action of the compound. The compound was devoid of cell toxicity up to 100 μg/mL against a reference A549 cell line. Likewise, RM33 did not induce apoptosis in these cells. The compound stimulated concanavalin A (ConA)-induced splenocyte proliferation but did not change the secondary humoral immune response in vitro to sheep erythrocytes. Nevertheless, a low suppressive effect was registered on lipopolysaccharide (LPS)-induced splenocyte proliferation and a stronger one on tumor necrosis factor alpha (TNFα) production by rat peritoneal cells. The analysis of signaling pathways elicited by RM33 in nonstimulated resident cells and cell lines revealed changes associated with cell activation. Most importantly, we demonstrated that RM33 enhanced production of cyclooxygenase 2 in LPS-stimulated splenocytes. Based on the previous and herein presented results, we conclude that RM33 is an efficient, nontoxic immune suppressor with prevailing anti-inflammatory action. Additionally, structural studies were carried out with the use of appropriate spectral techniques in order to unequivocally confirm the structure of the RM33 molecule. Unambiguous assignment of NMR chemical shifts of carbon atoms of RM33 was conducted thanks to full detailed analysis of ^1^H, ^13^C NMR spectra and their two-dimensional (2D) variants. Comparison between theoretically predicted chemical shifts and experimental ones was also carried out. Additionally, *N*-deuterated isotopologue of RM33 was synthesized to eliminate potentially disturbing frequencies (such as NH, NH_2_ deformation vibrations) in the carbonyl region of the IR (infrared) spectrum to confirm the presence of the carbonyl group.

## 1. Introduction

Compounds containing an isoxazole ring [1] constitute an important source of valuable drugs, designed to treat inflammatory states and diseases of different etiology. Syntheses, classification, mechanisms of action and therapeutic application of registered isoxazole derivatives, as well as these under preclinical investigations, were recently described in detail in several reviews [2,3,4].

Leflunomide and its active metabolites [5] are the most well-recognized immunosuppressive drugs which have found therapeutic applications if used alone or in combination with other drugs in rheumatoid arthritis [6] or transplantation [7]. Another example of a widely used isoxazole drug is Parecoxib, a pain reliever that acts as a cyclooxygenase 2 (COX-2) inhibitor [8,9]. A number of isoxazole compounds demonstrating immunosuppressive and anti-inflammatory properties have been under investigation at various stages of basic and preclinical studies [10,11,12,13,14,15,16,17,18,19,20,21,22,23].

The mechanisms of action of immunosuppressive isoxazoles of therapeutic utility may differ and include, among other things, COX-2 inhibition [5,8,19,24], increase in expression of caspases, Fas and NFκB1 [25], upregulation of IL-17F and downregulation of IL-10 and TLR4 expression [22], suppression of tumor necrosis factor alpha (TNFα), IL-1β, macrophage migration inhibition factor (MIF), NFκB and p38 expression [13], inhibition of lipopolysaccharide (LPS)-induced nitric oxide [18], TNFα and IL-6 production in macrophages [26], and fatty acid amide hydrolase activity in the inflamed gut [15].

Among immunosuppressive isoxazole derivatives synthesized by our research team, an isoxazolo[5,4-e]-1,2,4-triazepine derivative (denoted as RM33) has attracted special interest due to its decisive immunosuppressive properties in in vivo models with a potency compared with that of cyclosporine in inhibition of the immune response. We showed that RM33 administered intraperitoneally (i.p.) before immunization significantly decreased number of antibody forming cells to sheep red blood cells (SRBCs) in mice [27] and carrageenan-induced foot pad inflammation in rats [28]. The compound also inhibited an inductive and effectual phase of delayed type hypersensitivity to ovalbumin (OVA) [27]. RM33 was also effective when administered per os. A suppressive effect of RM33 in generation of the delayed type hypersensitivity to OVA could be also achieved when the compound was admixed with Freund’s complete adjuvant for a subcutaneous immunization of mice with OVA. RM33 also reduced serum level of TNFα induced by intravenous administration of LPS [28]. In vitro experiments, performed in parallel, unexpectedly revealed that RM33 did not inhibit proliferative response of mitogen-induced peripheral blood mononuclear cells and LPS-induced TNFα production.

This apparent discrepancy between in vivo and in vitro actions of RM33 prompted us to investigate the mechanism of action of the compound in a mouse in vitro model by determination of mitogen-induced splenocyte proliferation, secondary humoral immune response to SRBC and cytokine production by peritoneal cells. In addition, we investigated cytotoxic actions of the compound towards cell lines and cells from the lymphoid organs. In order to find a plausible mechanism of action of RM33, we analyzed effects of the compound on expression of signaling molecules in mouse bone marrow cells, thymuses and splenocytes, as well as cell lines representing major cell types such as T, B cells, macrophages and myelocytic precursors. In addition, we evaluated effects of RM33 on viability and apoptosis of resident cells from the lymphoid organs and J744 macrophage cell line. The function of macrophages was evaluated by RM33 effects on LPS-induced TNFα production in peritoneal exudates cell culture and pinocytic activity of J774 cell line. Finally, we evaluated effect of RM33 on the levels of cyclooxygenases in LPS-stimulated splenocytes. The biological part of the investigation is accompanied by description of RM33 synthesis and analysis of its identity and physicochemical properties by application of various spectroscopic techniques. It is important to note that the isoxazolo[5,4-e]-1,2,4-triazepine system has been so far little studied, both physicochemically and biologically [29].

## 2. Results

### 2.1. Chemistry

**Scheme 1:**
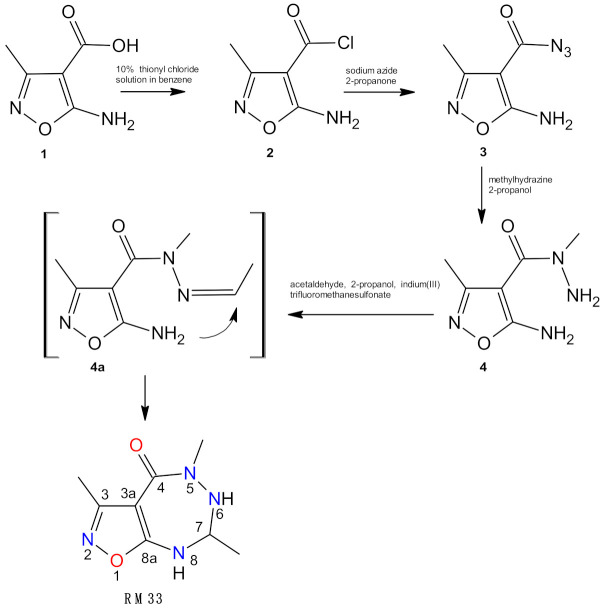
Synthesis of the 3,5,7-trimethyl-5,6,7,8-tetrahydro-4H-[1,2]oxazolo[5,4-e][1,2,4]triazepin-4-one (RM33).

For the first time, the synthesis of the isoxazolo[5,4-e]-1,2,4-triazepine system was presented in 1985 by Hasnaoui et al. [30], where an isoxazole ring was cyclized on an existing, appropriately substituted 1,2,4-triazepine ring with usage of appropriate nitrile N-oxide. Generally, this system can be obtained in two ways: by adding the isoxazole ring to the 1,2,4-triazepine system, as mentioned above, or by forming a 1,2,4-triazepine ring to the isoxazole ring [31]. 3,5,7-Trimethyl-5,6,7,8-tetrahydro-4H-[1,2]oxazolo[5,4-e][1,2,4]triazepin-4-one (RM33), used in experiments, was synthesized and purified with the following efficient method presented in Scheme 1.

5-Amino-3-methyl-4-isoxazolecarboxylic acid (1) [32] was obtained by hydrolysis process of its appropriate methyl or ethyl esters [33]. Initially, 5-amino-3-methyl-4-isoxazolecarbonyl chloride (2) and 5-amino-3-methyl-4-isoxazolecarboxylic acid azide (3) were obtained according to a previously described method by Ryng [34] and were used to obtain 5-amino-N,3-dimethyl-1,2-oxazole-4-carbohydrazide (4) and 3,5,7-trimethyl-5,6,7,8-tetrahydro-4H-[1,2]oxazolo[5,4-e][1,2,4]triazepin-4-one (RM33).

Hydrazide 4 was obtained in a reaction of 5-amino-3-methylisoxazole-4-carboxylic acid azide with methylhydrazine, according to the method described in [25]. The final RM33 was synthesized in two steps through intermediate product, i.e., N-acylhydrazone 4a, which was formed in the nucleophilic addition reaction of terminal NH_2_ group of hydrazide 4 to carbonyl carbon of acetaldehyde. In second step intermediate 4a was cyclized as a result of reaction of intramolecular nuclephilic addition of isoxazole amine group (aza-Michael type reaction) to hydrazone carbon catalyzed by indium (III) trifluoromethanesulfonate.

#### 2.1.1. Proof and Analysis of the Structure of RM33 Compound

The oxazolo[5,4-e]-1,2,4-triazepine system is relatively little known and only a few its derivatives have been described. As, up to date, no proof of a structure of the considered compound, RM33, has been conducted, it is necessary to present reliable research results confirming its structure. Firstly, the elemental analysis and MS spectrum of the investigated compound point out that its molecular formula is C_8_H_12_N_4_O_2_.

**Figure 1:**
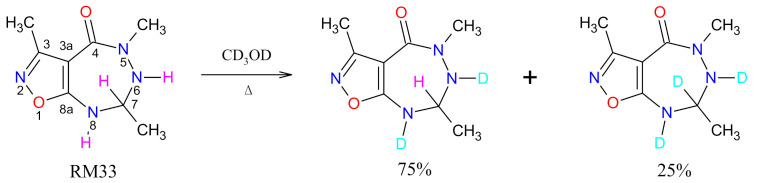
The *N*-deuteration of RM33 resulting in mixture of 6,8-dideuterio-3,5,7-trimethyl-7H-isoxazolo[5,4-e]-1,2,4-triazepin-4-one (about 75%) and 6,7,8-trideuterio-3,5,7-trimethyl-isoxazolo[5,4-e]-1,2,4-triazepin-4-one (about 25%).

In order to prove the presence of a carbonyl group in the considered molecule of RM33, its ATR FT-IR spectrum was recorded. This spectrum (Figure S1 in Supplementary Materials) shows that the strong band of the carbonyl region occurs at an extremely low wavenumber (1607 cm^−1^), as is the case for a carbonyl of tertiary cyclic amides (lactams). Assuming the presence of a carbonyl group in the tested compound, it should be taken into account that stretching vibration bands (mostly strong or of very strong intensity) of carbonyl groups in the IR spectrum can be often covered or perturbed with intensive bands of deformation vibrations of N-H (mainly scissoring vibration of NH_2_) groups. For example, 5-amino-3-methyl-4-isoxazolecarbohydrazide (without an *N*-methyl group), structurally similar to compound 4, exhibits the intense bending (scissoring) vibration of the NH_2_ amino group with a maximum at about 1616 cm^−1^ and a weak scissoring vibration of the terminal NH_2_ of the hydrazide group at about 1684 cm^−1^ [35]. Therefore, for this reason, the *N*-deuteratation of RM33 was carried out to remove potentially disturbing and covering bands to unambiguously assign the appropriate band to the carbonyl group (see Figure 1).

Generally, the results show that the structure of bands and the wavenumber values in the vibration region of the carbonyl groups do not change much after *N*-deuteration (the visualization of the spectra, Figures S2–S4). After deuteration, this band is still strong and visible with maximum at 1603 cm^−1^, and thus the red-shift of this band is slight, which proves that was not covered and disturbed by bending vibrations of bonds NH (NH_2_) and really coming from stretching vibration of carbonyl group. Additionally, stretching vibrations of the bonds between ring atoms of an aromatic isoxazole occur below 1600 cm^−1^, i.e., 1587 and 1564 cm^−1^, so they also cannot cover, disturb or be mistakenly taken for the considered carbonyl band. For comparison, for the 5-amino-3-methyl-4-isoxazolecarbohydrazide (without an *N*-methyl group) analogous to compound 4, they were observed at 1562 and 1523 cm^−1^ as very intense bands of stretching vibrations, C=N and C=C, respectively [35]. Therefore, the conclusion is that such a low frequency of stretching vibration of carbonyl group of considered compound RM33 is the result of strong coupling of this group with the aromatic system of the isoxazole ring through resonance effect in conjugated system. It additionally proves a low ring tension, which confirms the presence of seven membered ring of triazepine forming by indium(III) catalyzed cyclization step. However, *N*-deuteration of the RM33 made the disappearance of intense bands associated with NH bond stretching vibrations visible in the 3100–3400 cm^−1^ range, which are shifted to a lower frequency and appear as intense stretching vibrations of ND bonds in the 2300–2500 range due to the mass of deuterium being double that of a proton (Figures S2 and S3). The unchanged but better visible bands in the 2900–3000 cm^−1^ range (maxima at 2932 and 2988 cm^−1)^ after deuteration come from the C-H stretching vibrations of the methyl and methanetriyl (methin) groups.

**Figure 2:**
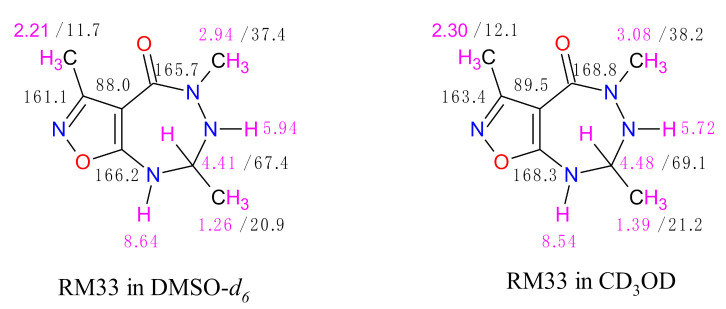
Experimental chemical shifts (ppm, values are marked for protons and carbons in pink and in black, respectively) in ^1^H and ^13^C NMR spectra of RM33 in various deuterated solvents.

Since standard one-dimensional (1D) ^1^H and ^13^C NMR spectroscopy is insufficient to unambiguously assign chemical shifts to the corresponding hydrogen and carbon atoms (Figure 2), two-dimensional (2D) NMR methods were used. Admittedly, on the basis of the ^1^HNMR spectrum in DMSO-*d*_6_ (see Figures S5A,B in Supplementary Materials), it can be assumed that there is a coupling between protons of the methyl group with a chemical shift of 1.26 ppm (broadened doublet, J ≈ 5 Hz) and a single proton with a chemical shift of 4.41 ppm (multiplet). However, due to the fact that the 4.41 ppm signal (multiplet) is heavily distorted and broadened, it is impossible to determine unquestionably its constant coupling, confirming the scalar coupling of this proton to the methyl group at 1.26 ppm (broadened doublet, J ≈ 5 Hz). Moreover, it is also impossible to clearly determine whether the protons with chemical shifts of 5.72 and 8.54 ppm are coupled to this proton at 4.41 ppm. For this reason, the spectrum of ^1^H-^1^H 2D-COSY45 was recorded, which dispels this doubt by indicating that they (i.e., N-H) are indeed scalar coupled to the considered proton (4.41 ppm, multiplet) (see Figure S9A in Supplementary Materials). It also confirms the scalar coupling between the protons of the methyl group at 1.26 ppm (broadened doublet) and the proton of the CH group (multiplet at 4.41 ppm). ^1^H-^13^C 2D HSQC spectroscopy, which reveals the coupling between protons and carbons through one bond, was used for correct assignment of chemical shifts of the carbon atoms directly bound to protons. The HSQC spectrum (see Figure S9B in Supplementary Materials) shows that carbon with a chemical shift 37.4 ppm is directly bound to three protons with a chemical shift of 2.94 ppm and it means that this methyl group is connected to nitrogen atom (position 5 of the system). In addition, the HSQC spectrum shows that the methyl group protons with a chemical shift of 1.26 ppm (position 7 of the system) are coupled to a carbon with a chemical shift of 20.9 ppm, which means that three protons (isoxazole methyl) with a chemical shift of 2.21 ppm are bound to a carbon with a shift of 11.7 ppm. Due to the fact that both protons connected to nitrogen atoms (N-H at positions 6 and 8 of the system) are scalarly coupled through three bonds with the proton from position 7, it is not possible to assign them unambiguously to the appropriate chemical shifts by means of the COSY45 spectroscopy. For this reason, the NOESY spectrum was recorded, which shows the spatial couplings through the space between distant protons (up to 4 Å). The NOESY spectrum showed that a proton with a chemical shift of 5.94 ppm is coupled with the protons (2.94 ppm) of the *N*-methyl group at position 5 of the system (see Figure S9C). On the other hand, there is no visible spatial coupling between the proton with chemical shift of 8.64 ppm and the protons of the *N*-methyl group, which means there is too large a spatial distance. Therefore, the conclusion is that the proton with chemical shift 5.94 ppm is a proton at position 6 of the system, which necessarily places the proton with a chemical shift of 8.64 ppm at position 8. In general, the proton at position 8 should be more acidic than the proton at position 6 due to the proximity of the oxygen atom of the isoxazole ring and an electron withdrawing effect through this ring. Additionally, the theoretically predicted chemical shifts for these protons indicate that the proton at position 6 will be more shielded by electrons than the proton at position 8 (see Section 2.1.2, Theoretical Prediction of Chemical shifts of ^1^H NMR and ^13^C NMR Spectra of RM33). To unambiguously assign quaternary carbon atoms to the appropriate values of their chemical shifts, the 2D ^1^H-^13^C Heteronuclear Multiple Bond Correlation (HMBC) method of NMR spectroscopy is particularly useful. In the HMBC spectrum (in DMSO-*d*_6_), long range couplings (through 2 to 4 bonds) between protons and carbons are visible (see Figure S9D):

- Strong long range coupling through two bonds between protons of methyl group (2.21 ppm) at position 3 of system and the ring carbon 3 (161.1 ppm);
- Slightly weaker through three bonds between protons of methyl group (2.21 ppm) at position 3 of system with the bridged ring carbon 3a (88.0 ppm);
- Very weak, but noticeable, through four bonds between protons of methyl group (2.21 ppm) at position 3 of system with the bridged carbon 8a (166.2 ppm);
- Long range coupling through three bonds between protons of methyl group (2.94 ppm) at position 5 of the system and the carbonyl carbon atom (165.7 ppm) are also clearly visible.

It should also be noted that the chemical shifts of the carbons of the carbonyl group and bridged carbon 8a of the isoxazolotriazepine system differ only by about 0.5 ppm and strongly depend on the solvent used. In the strongly polar protic methanol, the carbon nucleus of the carbonyl group is the most deshielded of all the carbon atoms of the isoxazolotriazepine system, and in the slightly less polar aprotic DMSO-*d*_6_, it is carbon 8a (Figure 2 and Figures S7, S8 and S9D,E in Supplementary Materials).

It is also worth mentioning that the proton bound to the carbon atom at position 7 (CH group) of the isoxazolotriazepine system is sufficiently acidic to be also partially exchanged (about 25%) into deuterium, causing the formation of a 6,7,8-trideuterio-3,5,7-trimethyl-isoxazolo[5,4-e]-1,2,4-triazepin-4-one during 48 h hydrogen–deuterium exchange reaction under influence of boiling deuterated methanol (CD_3_OD) (Figure 1). The visualizations of NMR spectra can be found in the Supplementary Materials (Figures S5–S11).

Finally, the HR-ESI-MS technique was used, the major advantage of which is that it is very selective since it measures the exact mass of a compound, allowing even minor changes in the structure to be distinguished. For RM33, a mass accuracy error was below 5 ppm (i.e., 0.736 ppm), which also confirms the assumed structure (see Section 4.1.1 in Materials and Methods).

#### 2.1.2. Theoretical Prediction of Chemical Shifts of ^1^H NMR and ^13^C NMR Spectra of RM33

For comparison, experimental chemical shifts with theoretically predicted appropriate calculations were carried out using methods described in detail in Section 4.1.3 and Section 4.2. The obtained results (Table 1) for proton chemical shift simulation indicate that the good agreement between the calculated chemical shifts and the experimental values is only for the protons bound to carbon atoms. The biggest discrepancy between theoretically predicted and experimental chemical shifts in the ^1^H NMR spectrum is for the N-H acidic protons. Their calculated electron shielding values are much bigger (i.e., lower chemical shifts) than the experimental values. Additionally, the calculated chemical shift for the nitrogen bonded proton in position 6 is smaller than for the carbon bonded proton in position 7, which is inconsistent with the experimental data, where it is the reverse. In turn, the experimental ^13^C NMR spectrum and theoretically predicted results consistently show that the carbon C7 atom is the most shielded by elelctrons (of course, not counting the carbons of the methyl groups) of all ring carbon atoms of isoxazolo[5,4-e]-1,2,4-triazepine system. In the aromatic isoxazole ring of the isoxazolotriazepine system, the quaternary bridged carbon C3a is the most shielded by electrons. It is caused by substantial contribution of resonance structures where high electron density appears, which is consistent with previous observations regarding 5-amino-3-methyl-4-isoxazolecarbohydrazide [35]. Additionally, the calculated chemical shifts for the ^13^C NMR spectrum show that the quaternary carbon of the carbonyl group is most deshielded, followed by the C3 quaternary and in the order of the bridged quaternary C8a carbon, which is inconsistent with the experimental (in DMSO-*d*_6_) results, which show that the most deshielded is C8a quaternary carbon, followed by the carbonyl, and the least is C3. However, in CD_3_OD, the most deshielded is carbonyl carbon, then C8a, and the least is C3. A very strong solvent effect can be seen here. This shows that, although predicted theoretical chemical shifts are useful in interpreting the NMR spectra, unambiguously certain assignments of chemical shifts to the respective atoms can only be achieved with appropriate NMR experimental techniques.

### 2.2. Activity of RM33 in Immunological Models In Vitro

Previous studies on RM33 activity in vivo (Table 2) revealed significant immunosuppressive and anti-inflammatory properties of the compound in several mouse and rat experimental models. These actions regarded both antigen-specific and nonspecific immune responses, of both humoral and cellular types. In addition, the compound affected both inductive and effectual phase of the immune response. The anti-inflammatory actions of RM33 were reflected by respective changes in cytokine release and amelioration of histopathological alterations.

**Figure 3:**
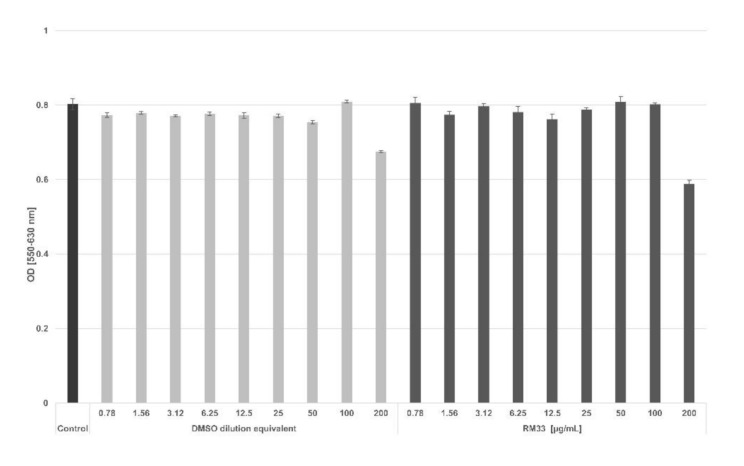
Cell toxicity of RM33 against reference A549 cell line. The test was performed in a monolayer culture of cell line A549 that were incubated at density 5 × 10^4^/well for 48 h. RM33 was tested at the 0.78–200 μg/mL concentration range. Control cultures contained corresponding dilutions of DMSO. The cell viability was determined by the MTT colorimetric assay. The results are presented as OD mean values ± SE from quadruplicate determinations.

In a preliminary experiment, RM33 was tested for its potential toxicity against a reference A549 cell line. Appropriate dilutions of the solvent (DMSO) served as controls. The results show no sign of toxicity up to 100 μg/mL concentration of the compound as evaluated by MTT colorimetric method and cell morphology (Figure S13). A small toxic effect of RM33 was only observed at 200 μg/mL concentration (Figure 3).

To determine effects of RM33 on metabolism and number of viable cells upon culture with RM33, the cells from mouse lymphatic organs were incubated overnight with 20 μg/mL of RM33 and cell viability was determined by MTT colorimetric method in comparison to appropriate DMSO control culture. The results (Table S1) revealed only a small (18.5%) decrease in viability of splenocytes, minor (1.8%) increase in viability of thymocytes and increase of 17.8% in viability of bone marrow cells.

**Figure 4:**
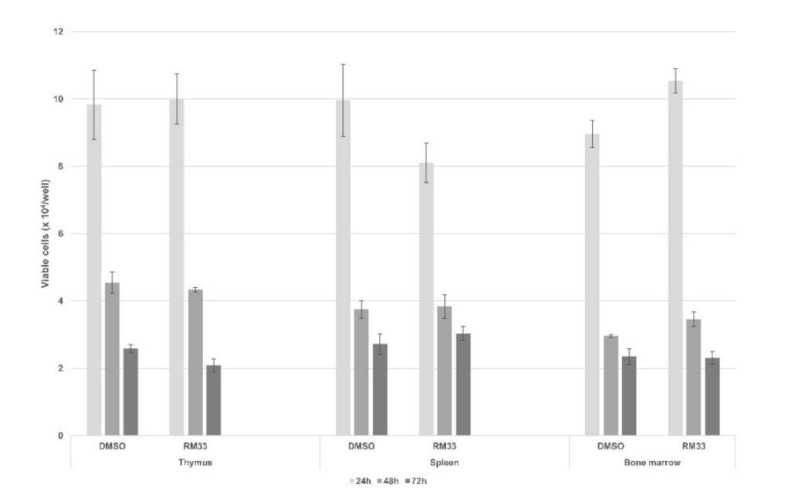
The effect of RM33 on the number of viable of cells from mouse lymphoid organs. Trypan blue dye exclusion method was applied. The number of viable (unstained) cells was determined in a Bürker hemocytometer. The results are presented as number of viable cells (mean values ± SE).

The enumeration of cells by trypan blue dye exclusion method in cell cultures of bone marrow cells, thymocytes and splenocytes in the same conditions after 24 h of culture led to similar results (Figure 4)—i.e., a small decrease in the number of splenocytes but respective increases in the number of thymocytes and bone marrow cells were observed. However, the number of splenocytes at 48 and 72 h were not negatively affected.

In addition, the results presented in Table 3 revealed no induction of apoptosis by the compound, at 2–50 µg/mL concentration range, with regard to these cell populations.

**Figure 5:**
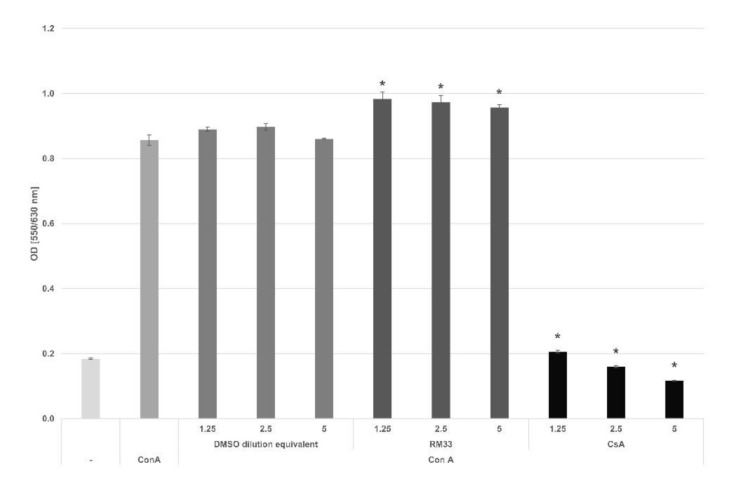
Effect of RM33 on ConA-induced mouse splenocyte proliferation. Splenocytes were distributed into tissue culture plates at a density of 2 × 10^5^/100 µL/well. An amount of 2.5 µg/mL ConA was added to induce cell proliferation. RM33 and CsA were added to the cultures at 1.25–5 µg/mL concentration range. After a 3-day incubation, the cell proliferation was determined using the colorimetric MTT assay [36]. The results are presented as the mean OD ± SE from quadruplicate determinations, * *p* < 0.05 (ANOVA; vs. DMSO dilution equivalent).

Figure 5 presents effects of RM33 on ConA-induced proliferation of splenocytes at a concentration range of 1.25–5 μg/mL. Cyclosporin A (CsA) served as a reference antiproliferative drug. The effects of RM33 were compared with appropriate concentrations of DMSO. The results showed stimulatory effects of RM33 in the investigated concentration range. A very strong suppressive effect was registered using CsA. Nevertheless, in some experiments, this stimulation was not registered (Table S2) and was absent with proliferation of human peripheral blood lymphocytes stimulated by PHA (Table S3).

**Figure 6:**
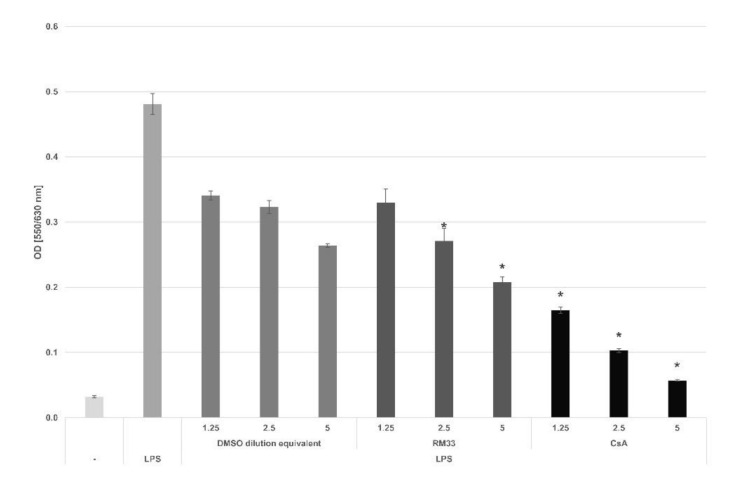
Effect of RM33 on LPS-induced mouse splenocyte proliferation. Splenocytes were distributed into 96-well flat-bottom tissue culture plates at a density of 2 × 10^5^/100 µL/well. LPS (50 µg/mL) was added to induce cell proliferation. RM33 and CsA were added to the cultures at 1.25–5 µg/mL concentration range. After a 3-day incubation, the cell proliferation was determined using the colorimetric MTT assay. The results are presented as the mean OD ± SE from quadruplicate determinations, * *p* < 0.05 (ANOVA; vs. DMSO dilution equivalent).

**Figure 7:**
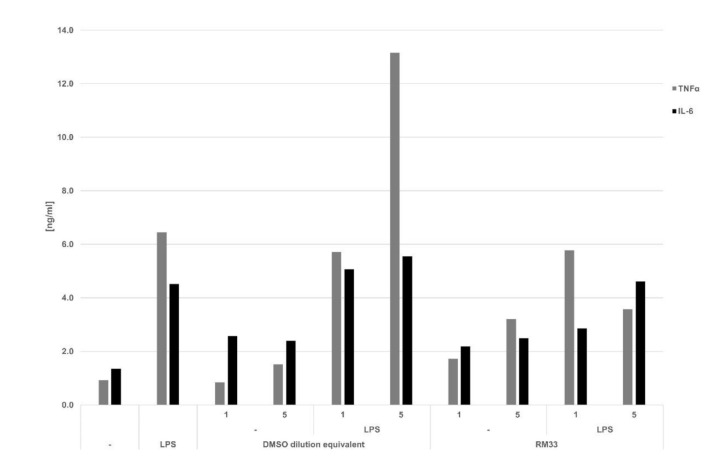
Effect of RM33 on LPS-induced cytokine production by rat peritoneal cells. Peritoneal cells were incubated overnight in 24-well culture plates containing 5 × 10^6^ cells/mL. LPS was applied at concentration of 1 µg/mL and RM33 at 1 and 5 µg/mL. Control cultures contained appropriate dilutions of DMSO, corresponding to DMSO dilutions at respective RM33 doses. The determination of TNFα and IL-6 was performed by bioassays [38,39]. The results are presented in ng/mL.

The compound did not affect the magnitude of the secondary humoral immune response to SRBC in vitro (Table S4). Nevertheless, RM33 moderately inhibited, in a dose dependent manner, LPS-induced splenocyte proliferation (Figure 6), where B cells are the targets [37]. In addition (Figure 7), the compound inhibited LPS-induced TNFα production by rat peritoneal cells at concentration of 5 µg/mL (3581 vs. 13,162 pg/mL). Of note, RM33 elevated the spontaneous production of TNFα by 2-fold. IL-6 production was not significantly affected.

The effects of the compound on expression of signaling molecules in mouse bone marrow cells, thymocytes and splenocytes are presented in Table 4 and Table 5. RM33 caused differential changes in the expression of MAP kinases in cells from the respective organs (Table 4). In bone marrow cells, significant changes included block of ERK1 and decreases in p38γ and p38δ subunit expression as well as significant increases in p38β and JNK expression. In the splenocytes, significant augmentation of signaling protein expression included all p38 subunits and JNK with an exceptional increase (256×) in p38δ. In the thymocytes, expressions of p38β and p38δ, as well JNK, were elevated.

In the bone marrow cells, the compound elevated expression of caspase 9 and Fas and moderately NFκB1 (Table 5). A stronger stimulation of caspases 9 and 3 was observed in thymocytes, accompanied by an increase in Bcl-2 and NFκB1 expression. In the splenocytes, RM33 induced comparable levels of caspases 3 and 9 as in thymocytes, and significant increases in expression of Fas and NFκB1.

Table 6 presents effects of the compound on expression of MAP kinases in Jurkat cells. The most notable changes were observed among expression of p38 subunits, i.e., a very deep inhibition of p38γ and to a lesser degree of p38α, and 3.7-fold elevation of the p38δ subunit. On the other hand, no changes (not shown) in expression of caspases 3, 7, 8, 9, Bcl2, Fas, NFκB1 and p53 were registered. The effects of RM33 on the expression of signaling molecules in macrophage J774 cell line were strikingly strong (Table 7). Among signaling molecules of the MAP kinase family, strong elevations in expression were observed for ERK-2, p38β, p38γ, p38δ and JNK proteins. Relatively lower increases were observed with regard to caspase 3, Bcl-2 and Fas molecules, except NFκB1, whose expression was strongly upregulated. Although effects of RM33 on the pinocytic activity of J774 cells were not significant (Figure S14), this effect seemed to be dose dependent—i.e., 100 µg/mL concentration of the compound inhibited and a concentration of 25 µg/mL tended to stimulate pinocytosis.

The changes in expression of signaling molecules in immature B cell WEHI 231 line and monocyte/neutrophil precursors HL-60 cell line were negligible and are shown in the Supplementary Materials (Tables S5–S7).

In the last stage of our investigations on the mechanism of action of RM33, the compound was added to the culture of splenocytes stimulated with 20 µg/mL of LPS and on day 3 of the incubation the cellular concentration of cyclooxygenase 2 was measured by ELISA assay. The results shown in Table 8 revealed a small increase in COX-2 level in nonstimulated control culture and a 38.9% increase in the culture of LPS-stimulated cells.

## 3. Discussion

In this report, we attempted to elucidate the intriguing phenomenon of divergent activities of RM33 in vivo and in vitro. RM33 demonstrated significant suppressive effects on the humoral and cellular response in vivo, as well as on inflammatory responses [27,28], but negligible actions on mitogen-induced proliferative response of splenocytes and secondary humoral immune response in vitro, as found in this investigation (Table S4). The discrepancies between strong immunosuppressive actions of RM33 in vivo and lack of significant effects on humoral immune response and lymphocyte proliferation in vitro suggested that the compound could act as a prodrug and should be metabolized in the liver to become effective, as in the case of leflunomide [5]. However, since RM33 inhibited in vitro LPS-inducible TNFα production and splenocyte proliferation and regulated cyclooxygenase 2 production, such a possibility could be excluded.

In the majority of experiments, we studied actions of RM33 at relatively low concentrations in nonstimulated cells and cell lines, so we did not expect increases in expression of signaling molecules associated with apoptosis. In fact, RM33 did not show cytotoxic effects on the reference cell line and resident cells from lymphatic organs. Eventually, the experiment with iodine propidine excluded a proapoptotic property of the compound (Table 3).

We consistently observed changes in expression of signaling molecules from MAP kinase family responsible for cell proliferation/differentiation or apoptosis, indicating antiapoptotic action of RM33. This was particularly evident in cases of low or lack of p38α expression, initiating a proapoptotic signal [40] and increases in p38β and p38δ, the molecules mediating cell activation or differentiation [41,42,43]. Therefore, the increases in p38δ expression in Jurkat cells, accompanied by the lack of changes in expression of caspases, Fas, NFκB1 and p53, involved in cell apoptosis (Table 5 and Table 6), could explain stimulation of ConA-induced splenocyte proliferation (Figure 5). In addition, the tremendous increases in NFκB1 expression in the macrophage J774 cell line (Table 7) seem to be correlated with stimulation of the spontaneous TNFα production in rat peritoneal cells (Figure 7) since TNF α production is dependent on NFκB1 expression [44]. At the same time, both the metabolism and pinocytic activity of J774 cells were not significantly affected (Figure S14, Table S8) demonstrating no negative effect of RM33 on cell function. On the other hand, relevant to the postulated anti-inflammatory RM33 action, rat peritoneal cells, stimulated with LPS, produced less TNFα upon treatment with RM33 (Figure 7). In addition, LPS-induced proliferation of B cells from mouse splenocytes was also inhibited (Figure 6). Thus, both actions of RM33, observed in cell cultures, support anti-inflammatory properties of the compound in vivo against proinflammatory stimuli, such as LPS, Freund’s complete adjuvant and carrageenan [27,28]. Of importance, all the studied proinflammatory stimuli [45,46,47] use both TLR2 and TLR4 receptors, respectively.

The suppressive property of RM33 on the humoral immune response to SRBC was limited only to the inductive phase in the in vivo system. The lack of its effects in vitro (Table S4) could be explained by differences in the experimental models, since in the model of the secondary immune response in vitro antigen-specific T and B cells are transferred from antigen-primed mice to the culture additionally immunized with the antigen. This model already expresses a low level SRBC-specific background response and antigen-specific B cells may act as both antigen presenting and accessory cells to develop a strong secondary immune response [48,49].

Although the inhibition by RM33 of LPS-induced IL-8 in human whole blood cultures was small (Table S9), the inhibition of IL-8 production could have a significance in the in vivo suppression of carrageenan-induced inflammation [28] by lowering neutrophil migration [50] to the site of inflammation. In the rat model of carrageenan-induced inflammation, IL-8 plays a major role [51] and the signaling pathway induced by carrageenan involves TLR4 [45], as in the case of LPS [47].

The effect of RM33 on cell signaling in bone marrow cells (Table 4 and Table 5) may be difficult to interpret since the composition of the bone marrow cell population is heterologous and contains pluripotential cells and a big reservoir of granulocytes and myelocytic precursors in rodents [52], where the HL-60 cell line could serve as a representative cell type. Therefore, the changes in expression of signaling molecules may represent a resultant effect of RM33 on various cell populations contained in the bone marrow.

In the case of HL-60 cells, the increases in expression of MAP kinases were modest, except ERK-2 (5.2× increase), and thus are not conclusive (Table S7). Nevertheless, the overall effect of RM33 on signaling pathways in bone marrow cells suggests cell activation, since all members of MAP kinases were strongly stimulated (ERK-2 16×, p38β 138× and JNK 31×) (Table 4). The changes in cell signaling were positively correlated with the increase in cell viability by 17.8% (Figure 4).

The discovery that RM33 affects production of COX-2 (Table 8) significantly contributed to understanding its immunosuppressive actions towards humoral and cellular immune responses in vivo [27] since PGE2 is involved in suppression of these responses [53]. Stimulation of COX-2 production by RM33 could also have a role in suppression of skin inflammatory reactions such as adjuvant inflammation or carrageenan reaction [28] where CCL27 chemokine is a mediator [54]. Nevertheless, at this stage of investigation, it is difficult to propose definite and complete mechanism of action of the compound.

In conclusion, given the anti-inflammatory properties of RM33, accompanied with the lack of cytotoxicity at high doses and its ability to regulate cyclooxygenase activities, the compound may find broad potential application in therapy of inflammatory disorders. Our unpublished data revealed a good potency of RM33, administered topically in ointment, in decreasing manifestations of contact sensitivity to oxazolone, indicating a possibility of treating skin inflammatory disorders.

## 4. Materials and Methods

### 4.1. Chemistry

Melting points were determined on the Büchi M560 melting point apparatus (BÜCHI Labortechnik AG, Meierseggstrasse 40, CH-9230 Flawil, Sankt Gallen, Switzerland) and were uncorrected. Thin-layer chromatography method (TLC) was applied to monitor the reaction progress as well as to confirm the purity of the obtained compounds. Polygram SIL G/UV254 plates (Mocherey-Nagel, Düren, Germany) for TLC were used, eluting medium was chloroform/methanol (9:1), detection of the compounds on the chromatograms was carried out with UV light. The Attenuated Total Reflectance IR (ATR-FT-IR) spectra (4000–450 cm^−1^) were recorded on a Nicolet iS50 FT-IR spectrophotometer (Thermo Fisher Scientific Inc., Waltham, MA, USA) using clean solid forms of the compounds (on the diamond crystal surface, 32 scans, resolution: 1 cm^−1^, measurement temperature: 20–25 °C). IR Spectra were recorded with ATR intensity correction. It is known that the relative intensity of bands in an ATR spectrum increases with wavelength, causing the distortion of relative peak intensities in comparison with the classical transmission experiment [55]. Omnic Specta software (Thermo Fisher Scientific Inc., Waltham, MA, USA) was used for IR spectra analysis. Baselines of all IR spectra were corrected with autocorrection (fit order 2) and then all the spectra were normalized. Frequencies are reported in cm^−1^. The samples were applied as solids. ^1^H NMR (300.15 MHz), ^13^C NMR (75.47 MHz, broadband full decoupling method), ^1^H-^1^H 2D Correlation Spectroscopy (COSY) NMR (both channels F2 and F1: 300.15 MHz), ^1^H-^13^C 2D COSY NMR (channel F2: 300.15 MHz, channel F1: 75.47 MHz), 2D ^1^H-^13^C HSQC (Heteronuclear Single Quantum Coherence = Heteronuclear Single Quantum Correlation), 2D ^1^H-^13^C Heteronuclear Multiple Bond Correlation (HMBC) and Nuclear Overhauser Effect Spectroscopy (NOESY) spectra were recorded with Bruker Fourier spectrometer (Bruker Analytische Messtechnik GmbH, Rheinstetten, Germany) using 5mm tubes, at concentration of about 20–30 mg of compound in 0.6 mL (about 0.1 M solution) of deuterated dimethyl sulfoxide (DMSO-*d*_6_, 99.5%) and methanol (CD_3_OD, 99.8%). Before NOESY measurement, the sample (25 mg/0.6 mL) was degassed after dissolving in DMSO-*d*_6_ in a vacuum desiccator equipped with drying agents (NaOH and anhydrous CaCl_2_) and then argonized. Chemical shifts were corrected relative to chemical shift of the solvents and are given in ppm units. Signal multiplicities are represented by the following abbreviations: s (singlet), d (doublet), m (multiplet). NMR spectra were analyzed with Mnova Suite Chemist 14.2.0—Academic Desktop Software (Mestrelab Research, S.L. Feliciano Barrera 9B—Bajo15706 Santiago de Compostela SPAIN). Mass spectrometry was performed on Bruker Daltonic ESI-Q-TOF apparatus in methanol (MeOH). Theoretical monoisotopic mass calculation, MS spectrum analysis and estimation of errors performed with Compass DataAnalysis 4.2 software (Bruker Daltonik GmbH, Bremen, Germany). All chemicals were purchased from commercial suppliers. The solvents were dried according to standard procedures. All the NMR, IR and MS measurements were carried out in the Laboratory of Elemental Analysis and Structural Research, Faculty of Pharmacy, Wroclaw Medical University, Poland.

#### 4.1.1. General Procedure for the Synthesis of 3,5,7-Trimethyl-5,6,7,8-Tetrahydro-4H-[1,2]Oxazolo[5,4-e][1,2,4]Triazepin-4-One—RM33

To 1 mmol of 5-amino-*N*,3-dimethyl-1,2-oxazole-4-carbohydrazide dissolved in 10 mL of 2-propanol, 5 mmol acetaldehyde and catalytic amount of indium(III) trifluoromethanesulfonate were added. Mixtures were stirred and heated in a boiling temperature (82 °C) for 9 h. At the end of the reaction (controlled in a TLC), the mixture was cooled. The solution was evaporated in vacuum from the mixture. The crude product, which separated out, was collected on a filter. The unrefined compound was purified by crystallization from methanol. As a result, a pure product was obtained. Yield—74%; m.p.—210.5–212 °C (dec.) (measured with LLG uniMELT 2 melting point apparatus, LLG), 211–213 °C (dec.) (measured with the Büchi M560 melting point apparatus). Elemental analysis for formula C_8_H_12_N_4_O_2_ of RM33, Calculated/found: %C—48.97/48.54, %H—6.16/6.27, %N—28.56/27.63. Elemental analysis points out that the compound crystallizes with the methanol molecules in the lattice. Elemental analysis for formula C_8_H_12_N_4_O_2_•1/5CH_3_OH of RM33, calculated/found: %C—48.52/48.54, %H—6.42/6.27, %N—27.65/27.63.

HR-ESI-MS (negative ionization in CH_3_OH): m/z[u/e] calculated for C_8_H_11_N_4_O_2_ [M-H]^−^ 195.088749, found 195.088606, hence accuracy error was below 5 ppm (i.e., err = 0.144 mDa, err = 0.736 ppm, mSigma = 10.5) (Figure S12); ATR-FTIR (T = 298 K): ν_max_ (cm^−1^) 1608 (CO stretching vibration) (Figure S1); ^1^H NMR (DMSO-*d*_6_, 300.15 MHz, T = 297.9 K): δ (ppm) 1.262 (d, J ≈ 5.1 Hz, 3H, CH_3_ at positon 7 of the isoxazolotriazepine system), 2.207 (s, 3H, CH_3_ at positon 3 of the system), 2.940 (s, 3H, *N*-CH_3_ at positon 5 of the system), 4.406 (m, 0.7H relative integral, CH of nondeuterated form, proton at position 7 of the system), 4.689 (m, 0.27H relative integral, CH of *N*-deuterioisotopologues, proton at position 7 of the system), 5.938 (d, J ≈ 8.1 Hz, about 1H, noticeable exchange H-D, NH, proton at position 6 of the system), 8.64 (slightly broadened singlet, about 1H but noticeable exchange H-D, NH proton at position 8 of the system) (Figure S5A,B); ^1^H NMR (CD_3_OD, 300.15 MHz, T = 298.7 K): δ (ppm) 1.390 (d, J ≈ 6 Hz, 3H, CH_3_ at positon 7 of the isoxazolotriazepine system), 2.303 (s, 3H, CH_3_ at positon 3 of the system), 3.082 (s, 3H, *N*-CH_3_ at positon 5 of the system), 4.486 (m, 0.73H, CH of *N*-deuterioisotopologues, proton at position 7 of the system), 5.726 (d, residual proton H as a result of fast H-D exchange, NH, proton at position 6 of the system), about 8.552 (barely visible signal, residual proton H as a result of fast H-D exchange, 1H, NH proton at position 8 of the system) (Figure S6); ^13^C NMR (DMSO-*d*_6_, 75.5 MHz, T = 300 K): δ (ppm) 11.7 (carbon of methyl group at position 3 of the system), 20.9 (carbon of methyl group at position 7 of the system), 37.4 (carbon of *N*-methyl group at position 5 of the system), 67.4 (C7 carbon at position 7 of the system), 88.0 (quaternary bridgehead carbon C3a of the system), 161.1 (quaternary C3 carbon at position 3 of the system), 165.7 (quaternary carbonyl carbon at position 4 of the system), 166.2 (quaternary C5 carbon at position 5 of the system) (Figure S7); ^13^C NMR (CD_3_OD, 75.5 MHz, T = 299.6 K): δ (ppm) 12.1 (carbon of methyl group at position 3 of the system), 21.2 (carbon of methyl group at position 7 of the system), 38.2 (carbon of *N*-methyl group at position 5 of the system), 69.1 (C7 carbon at position 7 of the system), 89.5 (quaternary bridgehead carbon C3a of the system), 163.4 (quaternary C3 carbon at position 3 of the system), 168.3 (quaternary C5 carbon at position 5 of the system), 168.8 (quaternary carbonyl carbon at position 4 of the system) (Figure S8).

#### 4.1.2. *N*-Deuteration of 3,5,7-Trimethyl-5,6,7,8-Tetrahydro-4H-[1,2]Oxazolo[5,4-e][1,2,4]Triazepin-4-One (RM33)

150 mg of RM33 was refluxed with 5 mL of deuterated methanol (CD_3_OD) in a moisture-proof flask for 48h. Upon cooling the solution to room temperature, the product crystallized, which was filtered off to obtain 45.1 mg of dry compound after thorough drying in a desiccator. M.p. = 197–200°C (dec.) (measured in sealed capillary with the Büchi M560 melting point apparatus). In order to confirm the exchange of two NH protons to *N*-D deuterium, the ^1^H NMR spectrum (in CD_3_OD) was performed, which confirmed the complete conversion of *N*-protons to *N*-deuteriums. Then, the ATR-FTIR spectrum (with ATR correction) of the isotopologue RM33D sample was measured. ATR-FTIR (T = 298 K): ν_max_ (cm^−1^) 1603 (CO stretching vibration) (Figures S2–S4).

^1^H NMR (CD_3_OD, 300.15 MHz, T = 298.6 K): δ (ppm) 1.618 (d, 3H, CH_3_ at position 7 of the isoxazolotriazepine system), 2.530 (s, 3H, CH_3_ at position 3 of the system), 3.310 (s, 3H, *N*-CH_3_ at position 5 of the system), 4.714 (m, 0.74H relative integral, CH, proton at position 7 of the system), 5.948 (0.04H relative integral, residual proton as a result of fast H-D exchange, NH, proton at position 6 of the system), 8.749 (0.06H relative integral, residual proton H as a result of fast H-D exchange, 1H, NH proton at position 8 of the system) (Figure S10).

^13^C NMR (CD_3_OD, 75.5 MHz, T = 299.6 K): δ (ppm) 12.1 (carbon of methyl group at position 3 of the isoxazolotriazepine system), 21.2 (carbon of methyl group at position 7 of the system, broadened signal due to coupling with neighboring deuterium atom), 38.2 (carbon of *N*-methyl group at position 5 of the system), 69.1 (C7 carbon at position 7 of the system broadened signal due to C-D coupling), 89.5 (quaternary bridgehead carbon C3a of the system), 163.4 (quaternary C3 carbon at position 3 of the system), 168.3 (quaternary C5 carbon at position 5 of the system), 168.8 (quaternary carbonyl carbon at position 4 of the system) (Figure S11).

#### 4.1.3. Computational Details about Theoretical Prediction of Chemical Shifts of ^1^H NMR and ^13^C NMR Spectra of RM33

In the beginning, the geometry optimization of RM33 and tetramethylsilane (TMS) molecules was performed on the basis of ab initio quantum mechanical DFT (density functional theory) method using the B3LYP [56,57] hybrid density functional with the following basis functions: Pople type basis sets augmented with diffuse functions [58,59,60], and Dunning’s correlation consistent basis set—augmented with diffuse functions aug-cc-pVTZ [61]. The Conductor-like Polarizable Continuum Model (CPCM) [62] was used for simulation of DMSO environment with Gaussian 16 default set of parameters (scaling factor for van der Waals radius for all atoms—i.e., alpha = 1.1). All the calculations were computed using the Gaussian 2016 revision C.01 software [63]. To possibly get most accurate approximation the following keywords, i.e., Fopt = (Tight,CalcAll), SCF = (Direct,VeryTight) and Integral(Grid = SuperFineGrid) were used in the calculations. The absence of imaginary wavenumbers in the result of the frequency calculations (CalcAll or Freq keywords) proved a stationary point found (a minimum on the potential energy surface). The calculations of isotropic chemical shielding for ^1^H and ^13^C nuclei and then chemical shifts were performed similarly to a way described in [64,65]. Isotropic chemical shielding of NMR were calculated using Gauge-Independent Atomic Orbital (GIAO) method [66] with following combinations functional/basis set: B3LYP/6-311+G(d,p), B3LYP/6-311++G(df,pd) and B3LYP/aug-cc-pVTZ with application of CPCM [61] with default prarameters for simulation of solvent environment. Following parameters were used nmr (All, PrintEigenvectors, mixed, Susceptibility). Nondeuterated DMSO, as the solvent was set and its appropriate parameters (such as electric permittivity) used instead of its deuterated form in CPCM calculations. TMS was set as a reference. Hence, chemical shifts were calculated for ^1^H and ^13^C NMR. The calculated shifts and comparison with experimental values are collected in Table 1. The results show that the least deviations between calculated and experimental values are obtained for combination of B3LYP functional and medium 6-31+G(d,p) basis set. Larger basis sets, such as 6-311++G(df,pd) and aug-cc-pVTZ, gave worse results in this case.

### 4.2. Biology

#### 4.2.1. Animals

Two-month-old BALB/c mice of both sexes delivered by Center of Experimental Medicine, Medical University, Białystok, Poland, and DBA/1 mice from the animal facility of the Institute of Immunology and Experimental Therapy in Wrocław, Poland, were used as donors of the lymphoid organs. The mice were kept in 12/12h light/dark conditions and given drinking tap water and commercial food ad libitum. Female Wistar rats, 3-month-old, derived from the animal facility of Wrocław Medical University, Poland. The animals were used only as organ donors what does not require approval of ethics committee according to Directive 2010/63/EU of the European Union Parliament and of the Council of 22 September 2010 on the protection of animals used for scientific purposes.

#### 4.2.2. Reagents

Cyclosporin A (CsA) (Sandimmun, Neoral^®^) in ampoules was from Sandoz, Basel, Switzerland, RPMI-1640 medium from Life Technologies, Inchinnan, UK, fetal calf serum (FCS) from Gibco, cremophor^®^ and lipopolysaccharide (LPS) from *Escherichia coli* O:111, concanavalin A (Con A), phytohemagglutinin A (PHA), ovalbumin (OVA) and bovine serum albumin (BSA), 3-[4,5-dimethylthiazol-2-yl]-2,5-diphenyltetrazolium bromide (MTT) and dimethylsulfoxide (DMSO) from Sigma-Aldrich (St. Louis, MO, USA). Sheep red blood cells (SRBCs) were supplied by Wrocław University of Life and Environmental Sciences (Wrocław, Poland). The sequences of primers used in the study are presented in Table S10. RM33 was synthesized as described in Section 4.1.1. RM33 was initially dissolved in DMSO (1 mg/100 µL) and subsequently in the culture medium.

#### 4.2.3. Cell Lines

J774E, a mouse, monocyte/macrophage cell line was obtained as a kind gift of Dr Philip Stahl (Washington University School of Medicine, St Louis, MO, USA). HL-60 (ATCC CCL-240), a human promyeloblast leukemia cells, WEHI 231 (ATCC, CRL-1702), a murine B cell line and Jurkat (ATCC, TIB-152), a human T cell line and A549 (ATCC CCL 185), an epithelial lung cancer cell line, were obtained from the cell line bank of the Institute of Immunology and Experimental Therapy (Wroclaw, Poland).

#### 4.2.4. Propagation of Cell Lines

J774E cells were maintained in a culture medium consisting of RPMI-1640 containing 10% FCS and antibiotics, at a density of 2 × 10^5^ cells/mL. HL-60 cells were maintained in Iscove’s Medium Dulbecco’s Medium containing 10% FCS and antibiotics, at a density of 1 × 10^5^ cells/mL. Jurkat cells were cultured in a culture medium consisting of RPMI-1640 and 10% FCS and antibiotics, at a density of 1 × 10^5^ cells/mL. WEHI 231 cells were cultured in Dulbecco’s Modified Eagle’s Medium (DMEM) and 10% FCS and antibiotics, at a density of 1 × 10^5^ cells/mL. The cell cultures were maintained at 37 °C in 5% CO_2_ and 95% humidified atmosphere.

#### 4.2.5. Cell Toxicity Test

RM33 was initially dissolved in DMSO, further dilutions of the compound were performed in RPMI-1640 medium supplemented with 2% FCS. RM33 was tested at 0.78–200 μg/mL concentration range. The evaluation of a potential cytotoxic action of RM33 was performed in a monolayer culture of epithelial lung cancer cell line A549. The cells at density of 5 × 10^4^/well were incubated for 24 h in a cell culture incubator. After the incubation, the culture supernatants were removed and to the monolayer cultures of cells appropriate dilutions of RM33 in the culture medium (200 μL/well) were added and incubated for additional 48 h. Control cultures contained corresponding dilutions of DMSO. The cell viability was determined by MTT colorimetric assay [36].

#### 4.2.6. Isolation of Cells from the Lymphoid Organs

Mice were sacrificed by cervical dislocation. Thymuses, spleens and femurs were isolated and placed in disposable Petri dishes containing sterile, ice-cold PBS. The cells were released from the lymphatic organs by passing them through a nylon mesh and separating by centrifugation (200× *g*). The cells were then collected, washed with ice-cold PBS supplemented with 1% BSA and re-suspended in culture medium at density of 1 × 10^6^ cells/mL. The viability of the cell suspension, determined by trypan blue dye exclusion assay, was 90–98%.

#### 4.2.7. Evaluation of Viability of Cells from the Lymphoid Organs

The cells, isolated as described above, were diluted 20× in 0.1% trypan blue and counted in a hemocytometer. The number of viable (unstained) cells was determined.

#### 4.2.8. Isolation of Peritoneal Exudates Cells and Cytokines Determination

Rats were sacrificed in a CO_2_ atmosphere. The peritoneal cavities were washed with 20 mL of cold Hanks’ medium containing 5 units/mL of heparin. The cells were centrifuged at 200× *g* for 10 min, washed once with Hanks’ medium and counted in a hemocytometer. The cells were re-suspended in the culture medium in 1 mL aliquots in 24-well culture plates (Nunc) containing 5 × 10^6^ cells/mL. The cells were incubated overnight. LPS was applied at concentration of 1 µg/mL and RM33 at 1 and 5 µg/mL. Control cultures contained appropriate dilutions of DMSO, corresponding to DMSO dilutions at respective RM33 doses. Determination of TNFα and IL-6 were performed by bioassays [38,39].

#### 4.2.9. Proliferation Tests

The spleens were pressed against a plastic screen into 0.83% NH_4_Cl solution to lyze erythrocytes (5 min incubation at room temperature). The cells were then washed twice with Hanks’ medium, passed through a glass wool column to remove debris, and re-suspended in the culture medium, referred to below as the culture medium, consisting of RPMI-1640, supplemented with 10% FCS, L-glutamine, sodium pyruvate, 2-mercaptoethanol and antibiotics. The cells were then distributed into 96-well flat-bottom tissue culture plates (Nunc) at a density of 2 × 10^5^/well. 2.5 µg/mL ConA was added to induce cell proliferation. To induce B-cell proliferation LPS at concentration of 50 µg/mL was applied. RM33 was added to the cultures at doses of 1.25, 2.5 and 5 µg/mL. After a 3-day incubation, the cell proliferation was determined using the colorimetric MTT assay (see Section 4.2.10). The results are presented as the mean optical density (OD) at 550/630 nm ± standard error (SE) from quadruplicate determinations.

#### 4.2.10. Colorimetric MTT Assay

The cell proliferation was determined using the colorimetric MTT assay according to Hansen et al. [36]. Briefly, 25 µL of 3-[4,5-dimethylthiazol-2-yl]-2,5-diphenyltetrazoliumbromide (MTT, Sigma-Aldrich, St. Louis, MO, USA). From stock solution (5 mg/mL), 25 μL was added per well at the end of cell incubation period and the plates were incubated for additional 3 h in a cell culture incubator. Then, 100 µL of the extraction buffer (20% SDS with 50% DMF, pH 4.7) was added. After an overnight incubation, the OD was measured at 550 nm with the reference wavelength of 630 nm.

#### 4.2.11. Determination of Apoptosis

Cells from the lymphoid organs were isolated as described above and placed in 24-well plates at a concentration of 10^6^/well in the culture medium. The cells were cultured overnight with RM33 (2, 10 or 50 μg/mL) and appropriate dilutions of the solvent (DMSO). Next, the nonadherent cells were transferred to FACS tubes and the adherent cells were treated for 30 min with 100 μL of 0.1% trypsin solution containing EDTA, pH 8.0, to regain remaining adherent cells. Adherent cells were then washed with PBS containing 2.5% FCS, centrifuged and added to nonadherent cells, centrifuged, and the cell pellets were eventually re-suspended in 1 mL of PBS containing 2.5% FCS and placed in an ice bath. For DNA fragmentation evaluation, cells were fixed with 70% ethanol (POCh, Gliwice, Poland) and subsequently stained with propidium iodide (50 µg/mL) and RNA-se (0.02 mg/mL) (Sigma-Aldrich) as described [67].

#### 4.2.12. Total RNA Isolation

Total RNA isolation was performed with TRIzol Reagent (Ambion) accordingly to manufacturer’s recommendations. The cell pellet (2 × 10^6^ cells) was re-suspended in 1 mL of TRIzol reagent, shaken, incubated for 10 min at room temperature (RT), supplemented with 0.2 mL of chloroform, shaken vigorously for 15 s, incubated for 3 min at RT and centrifuged at 12,000× *g* for 15 min at 4 °C. The water phase was collected, transferred to a new tube, supplemented with 0.5 mL of isopropanol, incubated at RT for 10 min and centrifuged at 12,000× *g* for 10 min at 4 °C. The RNA pellet was washed with 1 mL of 75% ethanol, dried in air and dissolved in 20–30 μL of sterile diethylpyrocarbonate-treated Mili-Q water. RNA samples were stored at −20 °C.

#### 4.2.13. Reverse Transcription

Single stranded complementary DNA (cDNA) was synthesized with oligo (dT)12-18 primers from 5 μg of total RNA using Novazym VerteKit, according to the manufacturer’s instruction. The list of primers used in the study is presented in Table S10.

#### 4.2.14. Quantitative Analysis of Gene Expression by Real Time PCR

The expression of studied genes, i.e., caspases 3, 7, 8 and 9, Bcl-2, Fas, NFκB1 and p53, was determined with AmpliQ 5× HOT EvaGreen^®^ qPCR Mix Plus (noROX) (Novazym). The change in expression of each particular gene in samples treated with RM33 was calculated by comparison to such expression in control culture after earlier normalization of total mRNA content by GADPH expression.

#### 4.2.15. Determination of Cyclooxygenases in LPS-Stimulated Mouse Splenocytes

Splenocytes from DBA/1 mice were incubated in the culture medium for 72 h in 24-well culture plates and a density of 5 × 10^6^/mL. The cells were stimulated with LPS (20 µg/mL) and RM33 was used at 10 µg/mL. The control cultures contained concentration of DMSO in the culture medium corresponding to the concentration of DMSO in cultures containing RM33.

After the incubation, the cells were detached by treatment with 0.1% trypsin solution for 10 min followed by 3× wash in PBS. The dry cell pellets were suspended in 0.5 mL of lysis buffer consisting of 1 mM EDTA, 0.5% Triton X-100 and containing 10 mg/mL leupeptin, 10 mg/mL pepstatin, and 3 mg/mL aprotinin in PBS, pH 7.4. Lysis was carried out on the ice for 15 min. Lysates were preserved by being deep-frozen (−80 °C). Before use, the lysate samples were centrifuged at 2000× *g* for 5 min, and the supernatant was transferred to a new tube. Sample total protein concentration was determined by the spectrophotometric method for equilibrating lysates dilutions. The content of cyclooxygenases in cells was determined by ELISA kits (Human/Mouse Total COX-2 DuoSet IC ELISA, R&D Systems, Minneapolis, MN, USA) according to the manufacturer’s instructions.

#### 4.2.16. Statistics

Each experimental group consisted of four wells (determinations) for in vitro tests. The results were subjected to statistical analysis using analysis of variance (one-way ANOVA) in STATISTICA 7 for Windows. Brown–Forsyth’s test was used to determine the homogeneity of variance between groups. Due to nonconstant variance, the data were analyzed using the nonparametric the Kruskal–Wallis’ analysis of variance, followed by Dunn’s test to estimate the significance of the difference between groups. Significance was determined at *p* < 0.05. The results are presented as mean values ± SE.

## Figures and Tables

**Table 1:** Comparison of experimental and calculated ^1^H and ^13^C NMR chemical shifts (δ/ppm) of RM33 in DMSO-*d*_6_ environment. Deviation (Δδ) is difference between experimental value and theoretically predicted one.

Method	^1^H NMR		^13^C NMR	
	Chemical Shift δ (ppm)	Deviation Δδ (ppm)	Chemical Shift δ (ppm)	Deviation Δδ (ppm)
Experimental * ± SD **	1.26 ± 0.009 (CH_3_)	-	11.6 ± 0.13 (CH_3_) 20.8 ± 0.13 (CH_3_) 37.3 ± 0.15 (*N*-CH_3_) 67.4 ± 0.01 (C7) 88.0 ± 0.014 (C3a) 161.1 ± 0.01 (C3) 165.8 ± 0.06 (CO) 166.2 ± 0.02 (C8a)	- - - - - - - -
	2.21 ± 0.00 (CH_3_)	-		
	2.94 ± 0.00 (CH_3_)	-		
	4.41 ± 0.013 (CH)	-		
	5.94 ± 0.015 (NH)	-		
	8.64 ± 0.002 (NH)	-		
B3LYP/6-31+G(*d*,*p*)	1.36 (CH_3_)	−0.10	15.66 (CH_3_) 22.55 (CH_3_) 38.19 (*N*-CH) 72.40 (C7) 90.77 (C3a) 163.82 (C3) 163.94 (CO) 161.79 (C8a)	−4.06 –1.75 –0.89 −5.00 −2.77 −2.72 1.85 4.41
	2.28 (CH_3_)	−0.07		
	3.07 (CH_3_)	−0.13		
	4.70 (CH)	−0.29		
	3.79 (NH)	2.15		
	5.58 (NH)	3.06		
B3LYP/6-311++G(*df*,*pd*)	1.41 (CH_3_)	−0.15	15.76 (CH_3_) 23.64 (CH_3_) 39.70 (*N*-CH) 75.73 (C7) 94.80 (C3a) 173.94 (C3) 175.26 (CO) 173.42 (C8a)	−4.16 −2.84 −2.40 −8.13 −6.80 −12.84 −9.46 −7.23
	2.34 (CH_3_)	−0.13		
	3.08 (CH_3_)	−0.14		
	4.66 (CH)	−0.25		
	3.63 (NH)	2.31		
	5.52 (NH)	3.12		
B3LYP/aug-cc-pVTZ	1.40 (CH_3_)	−0.14	15.85 (CH_3_) 23.30 (CH_3_) 38.68 (*N*-CH_3_) 75.30 (C7) 95.27 (C3a) 174.20 (C3) 175.44 (CO) 173.22 (C8a)	−4.25 −2.50 −1.38 −7.90 −7.27 −13.08 −9.64 −7.01
	2.34 (CH_3_)	−0.13		
	3.09 (CH_3_)	−0.15		
	4.74 (CH)	−0.33		
	3.78 (NH)	2.16		
	5.67 (NH)	2.97		

* Acquisition and process parameters used during recording ^1^H and ^13^C NMR spectra are included in Supplementary Materials; experimental chemical shifts were corrected relative to chemical shift of the solvent; ** SD—the standard deviations of the chemical shifts were estimated by measuring the NMR spectra of three independent samples of the compound under the same conditions.

**Table 2:** The summary of effects of RM33 on innate and adaptive immune responses in vivo.

Model	Effects of RM33 Administration	Reference
Humoral immune response	Inhibition of the inductive phase	[27]
Delayed type hypersensitivity	Inhibition of the inductive and effectual phase	[27]
Edema induced by complete Freund’s adjuvant	Decrease in footpad edema	[28]
LPS-induced endotoxemia	Significant inhibition of serum TNFα, small inhibitory effect on IL-6 and no effect on IL-10 serum level	[28]
Carrageenan reaction	Decreased serum TNFα, reduced: mast cell and macrophage infiltration and edema of the connective tissue	[28]

**Table 3:** Effect of RM33 on apoptosis of cells from mouse lymphoid organs. Cells isolated from the lymphoid organs were cultured 106/well overnight with RM33 (2, 10 or 50 μg/mL) and appropriate dilutions of DMSO. For DNA fragmentation evaluation cells were stained with propidium iodide (50 µg/mL) and analyzed by FACS. The results are presented as increase in % of apoptotic cells vs. DMSO control.

Organ	Increase in % of Apoptotic Cells		
	RM33 (μg/mL)		
	2	10	50
Bone marrow	0	0	0
Thymus	1	3	6
Spleen	1	1	1

**Table 4:** Effect of RM33 on expression of MAP kinases in the mouse lymphoid organs. Cells from the organs were incubated overnight with 10 µg/mL of RM33. Total RNA isolation, reverse transcription and quantitative analysis of gene expression by Real Time PCR were performed as described in the Materials and Methods. Glyceraldehyde-3-phosphate dehydrogenase (GAPDH) was used as a housekeeping gene to normalize values obtained for the target genes. The values presented in the table reflect relative fold changes in response of the cells to RM33 compound, when the GAPDH gene expression was a control and assumed as equal to one.

Organ	ERK-1	ERK-2	p38α	p38β	p38γ	p38δ	JNK
Bone marrow	0.05	16.6	7.8	138.0	0.2	0.4	31.4
Thymus	2.7	6.7	11.9	31.7	4.5	70.9	92.8
Spleen	7.9	5.4	18.3	27.0	19.3	256.8	70.1

**Table 5:** Expression of signaling molecules in cells from lymphoid organs. The experiment was performed as described above.

Organ	Casp-3	Casp-8	Casp-9	Bcl-2	Fas	NFκB1
Bone marrow	2.0	0.0	23.1	1.9	19.2	4.8
Thymus	9.5	2.3	222.1	18.2	5.7	10.7
Spleen	5.4	1.5	198.9	0.8	22.2	8.8

**Table 6:** Effect of RM33 on expression of MAP kinases in Jurkat cells. Cells (0.5 × 10^6^/mL) were incubated overnight with 10 µg/mL of RM33 or appropriate dilution of DMSO (corresponding to the concentration of DMSO in cultures containing RM33).

	ERK-1	ERK-2	JNK	p38α	p38β	p38γ	p38δ
RM33	1.7	0.8	0.9	0.3	0.5	0.1	3.7

**Table 7:** Effect of RM33 on expression of signaling molecules in macrophage J774 cell line.

ERK-1	ERK-2	p38α	p38β	p38γ	p38ő	JNK	Casp-3	Casp-8	Casp-9	NFκB1	Bcl-2	Fas
0	5487	0	4629	1303	1177	902	295	1.46	0	3656	344	753

**Table 8:** Effect of RM33 on COX-2 level in mouse splenocyte cultures. Splenocytes were incubated (5 × 10^6^/mL) in the culture medium for 72 h. The cells were stimulated with LPS (20 µg/mL) and RM33 was used at 10 µg/mL. The control cultures contained appropriate concentration of DMSO in the culture medium.

Culture	Cox-2 (pg/mL)
Control	456
RM33	470
LPS	742
LPS + RM33	1031

## Data Availability

The data presented in the current study are available from the corresponding author upon reasonable request.

## Supplementary Materials

Visualizations of ESI-MS, IR and NMR spectra and other additional data associated with this paper can be found in the Supplementary Materials file (pdf) available online at https://www.mdpi.com/article/10.3390/ph14050468/s1, Figure S1. ATR-FT-IR (with ATR correction) of RM33, Figure S2. ATR-FT-IR (with ATR correction) spectrum of N-deuterated isotopologue of RM33, Figure S3. Superimposed ATR-FT-IR spectra of RM33 (red) and its N-deuterated isotopologue RM33D (blue), Figure S4. Superimposed ATR-FT-IR spectra (carbonyl region) of RM33 (red) and its N-deuterated isotopologue RM33D (blue), Figure S5. 1H NMR (in DMSO-d6) of RM33, Figure S5A. 1H NMR (in DMSO-d6) of RM33, Figure S6. 1H NMR (in CD3OD) of RM33. Spectrum was performed after ten minutes after dissolvation of RM33 in CD3OD, Figure S7. 13C NMR (in DMSO-d6) of RM33, Figure S8. 13C NMR (in CD3OD) of RM33, Figure S9A. 1H-1H 2D-COSY45 spectrum (in DMSO-d6) of RM33, Figure S9B. 1H-13C 2D HSQC spectrum (in DMSO-d6) of RM33, Figure S9C. NOESY spectrum (in DMSO-d6) of RM33, Figure S9D. 2D 1H-13C HMBC spectrum (in DMSO-d6) of RM33, Figure S9E. 2D 1H-13C HMBC spectrum (in CD3OD) of RM33, Figure S10. 1H NMR (in CD3OD) of N-deuterated isotopologue (RM33D) of RM33, Figure S11. 13C NMR (in CD3OD) of N-deuterated isotopologue (RM33D) of RM33, Figure S12. HR-ESI-MS (negative ionization in CH_3_OH) of RM33, whole spectrum (top), magnification of quasi molecular ion [M-H]- peak region (below), next to quasi-molecular ion (100% relative intensity), two small isotope quasi-molecular ion peaks are visible, i.e. 196.091418 (8.50%) and 197.093193 (0.70% relative intensity), Figure S13. Phase contrast image of A549 cells treated with RM33 at 100 μg/mL and 200 μg/mL, Figure S14. Determination of endocytic activity of J774 cell line incubated with RM33, Table S1. The viability of cells from lymphatic organs measured by the MTT colorimetric method, Table S2. Effect of RM33 on ConA-induced mouse splenocyte proliferation, Table S3. Effect of RM33 on PHA-induced human PBMC proliferation, Table S4. Effect of RM33 on the secondary humoral immune response to SRBC in vitro, Table S5. Effect of RM33 on expression of signaling molecules in WEHI 231 cells, Table S6. Effect of RM33 on expression of MAP kinases in WEHI 231 cells, Table S7. Effect of RM33 on expression of signaling molecules in promyelocytic HL-60 cell line, Table S8. Effect of RM33 (at indicated concentrations) on metabolism of J774 macrophage cell line, Table S9. Effect of RM33 on LPS-induced IL-8 production in human whole blood cell cultures, Table S10. The sequences of primers used in the study.
