# Supplementary material for: Synthesis, Physicochemical Characteristics and Plausible Mechanism of Action of an Immunosuppressive Isoxazolo[5,4-e]-1,2,4-Triazepine Derivative (RM33)
Source: Pharmaceuticals (Basel). 2021 May 15;14(5):468. doi: 10.3390/ph14050468 (PMC8156388; doi:10.3390/ph14050468)
Supplement: Supplementary file 1 [file pharmaceuticals-14-00468-s001.zip › pharmaceuticals-1202245-supplementary.pdf]

Supplementary Material

Synthesis, physicochemical characteristics and plausible mechanism of action of an immunosuppressive isoxazolo[5,4-e]-1,2,4-triazepine derivative (RM33)

Marcin Mączyński[1*], Andrzej Regiec[1], Aleksandra Sochacka-Ćwikła[1], Iwona Kochanowska[2], Maja Kocięba[2], Ewa Zaczyńska[2], Jolanta Artym[2], Wojciech Kałas[2] and Michał Zimecki[2]

[1] Wroclaw Medical University, Faculty of Pharmacy, Department of Organic Chemistry, 211A Borowska Street, 50-556 Wroclaw, Poland; marcin.maczynski@umed.wroc.pl (M.M); andrzej.regiec@umed.wroc.pl (A.R.); Aleksandra.sochacka-cwikla@umed.wroc.pl (A.S-Ć.)
[2] Hirszfeld Institute of Immunology and Experimental Therapy, Polish Academy of Sciences, Laboratory of Immunobiology, Weigla 12, 53-114 Wroclaw, Poland; maja.kocieba@hirszfeld.pl (M.K.); ewa.zaczynska@hirszfeld.pl (E.Z.); iwona.kochanowska@hirszfeld.pl (I.K.); jolanta.artym@hirszfeld.pl (J.A); wojciech.kalas@hirszfeld.pl (W.K.); michal.zimecki@hirszfeld.pl (M.Z.)
*Correspondence: marcin.maczynski@umed.wroc.pl; Tel.: +48717840340

TABLE OF CONTENTS

1. Chemistry **4**

1.1. Detailed analysis of ESI-MS spectra, visualizations of IR and NMR spectra. **4**

Figure S1. ATR-FT-IR (with ATR correction) of RM33 4
Figure S2. ATR-FT-IR (with ATR correction) spectrum of *N*-deuterated isotopologue of RM33 4
Figure S3. Superimposed ATR-FT-IR spectra of RM33 (red) and its *N*-deuterated isotopologue RM33D (blue) 5
Figure S4. Superimposed ATR-FT-IR spectra (carbonyl region) of RM33 (red) and its *N*-deuterated isotopologue RM33D (blue) 5
Figure S5. ^{1}H NMR (in DMSO-*d6*) of RM33 6
Figure S5A. ^{1}H NMR (in DMSO-*d6*) of RM33 7
Figure S6. ^{1}H NMR (in CD_3OD) of RM33. Spectrum was performed after ten minutes after dissolvation of RM33 in CD_3OD 8
Figure S7. ^{13}C NMR (in DMSO-*d6*) of RM33 9
Figure S8. ^{13}C NMR (in CD_3OD) of RM33 10
Figure S9A. ^{1}H-^{1}H 2D-COSY45 spectrum (in DMSO-*d6*) of RM33 11
Figure S9B. ^{1}H-^{13}C 2D HSQC spectrum (in DMSO-*d6*) of RM33 12
Figure S9C. NOESY spectrum (in DMSO-*d6*) of RM33 12
Figure S9D. 2D ^{1}H-^{13}C HMBC spectrum (in DMSO-*d6*) of RM33 13
Figure S9E. 2D ^{1}H-^{13}C HMBC spectrum (in CD_3OD) of RM33 13
Figure S10. ^{1}H NMR (in CD_3OD) of *N*-deuterated isotopologue (RM33D) of RM33 14
Figure S11. ^{13}C NMR (in CD_3OD) of *N*-deuterated isotopologue (RM33D) of RM33 14
Figure S12. HR-ESI-MS (negative ionization in CH_3OH) of RM33, whole spectrum (top), magnification of quasi molecular ion $[M-H]^-$ peak region (below), next to quasi-molecular ion (100% relative intensity), two small isotope quasi-molecular ion peaks are visible, i.e. 196.091418 (8.50%) and 197.093193 (0.70% relative intensity) 15

2. Biology **15**

2.1. Determination of RM33 toxicity by morphologic examination by microscopy **15**

Figure S13. Phase contrast image of A549 cells treated with RM33 at 100 μg/mL and 200 μg/mL. Cell growth, morphology and viability were used as parameters to determine the cytotoxicity of the compound 16

2.2. Effects of RM33 on viability of mouse lymphocytes and proliferation of mouse and human lymphocytes to mitogens **17**

Table S1. The viability of cells from lymphatic organs measured by the MTT colorimetric method 17
Table S2. Effect of RM33 on ConA-induced mouse splenocyte proliferation 17
Table S3. Effect of RM33 on PHA-induced human PBMC proliferation, *versus control, $p<0.05$ 18

2.3. Effect of RM3 on the secondary humoral immune response *in vitro* to SRBC **18**

Table S4. Effect of RM33 on the secondary humoral immune response to SRBC *in vitro*, *versus DMSO control, $p<0.05$ 18

2.4. Effects of RM33 on expression of signaling molecules in cell lines corresponding to immature B cells and granulocyte/monocyte precursors **18**

Table S5. Effect of RM33 on expression of signaling molecules in WEHI 231 cells 19
Table S6. Effect of RM33 on expression of MAP kinases in WEHI 231 cells 19
Table S7. Effect of RM33 on expression of signaling molecules in promyelocytic HL-60 cell line 19

2.5. Effects of RM33 on metabolism and pinocytic activity of J774 macrophage cell line **19**

Table S8. Effect of RM33 (at indicated concentrations) on metabolism of J774 macrophage cell line, *versus DMSO control, $p<0.05$ 19
Figure S14. Determination of endocytic activity of J774 cell line incubated with RM33 at indicated concentrations 20

2.6. Effect of RM33 on IL-8 production by human blood cells **20**
Table S9. Effect of RM33 on LPS-induced IL-8 production in human whole blood cell cultures; *versus DMSO control, $p < 0.05$ 20
2.7. The sequences of primers used in the study **21**
Table S10. The sequences of primers 21

3. Materials and Methods **22**
3.1. Preparation of RM33 solutions for *in vitro* studies **22**
3.2. Determination of RM33 toxicity **22**
3.3. Isolation of human PBMC **22**
3.4. Proliferation of human PBMC to phytohemagglutinin (PHA) **22**
3.5. Proliferation of mouse splenocytes to concanavalin A (ConA) **22**
3.6. Colorimetric MTT assay for cell growth and kill **23**
3.7. Secondary immune response to SRBC *in vitro* **23**
3.8. Effect on metabolism and endocytosis activity of J774 macrophage cell line **23**
3.9. Induction and determination of IL-8 in human whole blood culture **23**
3.10. Statistics **24**

References **24**

1. Chemistry

1.1. Detailed analysis of ESI-MS spectra, visualizations of IR and NMR spectra and other additional data

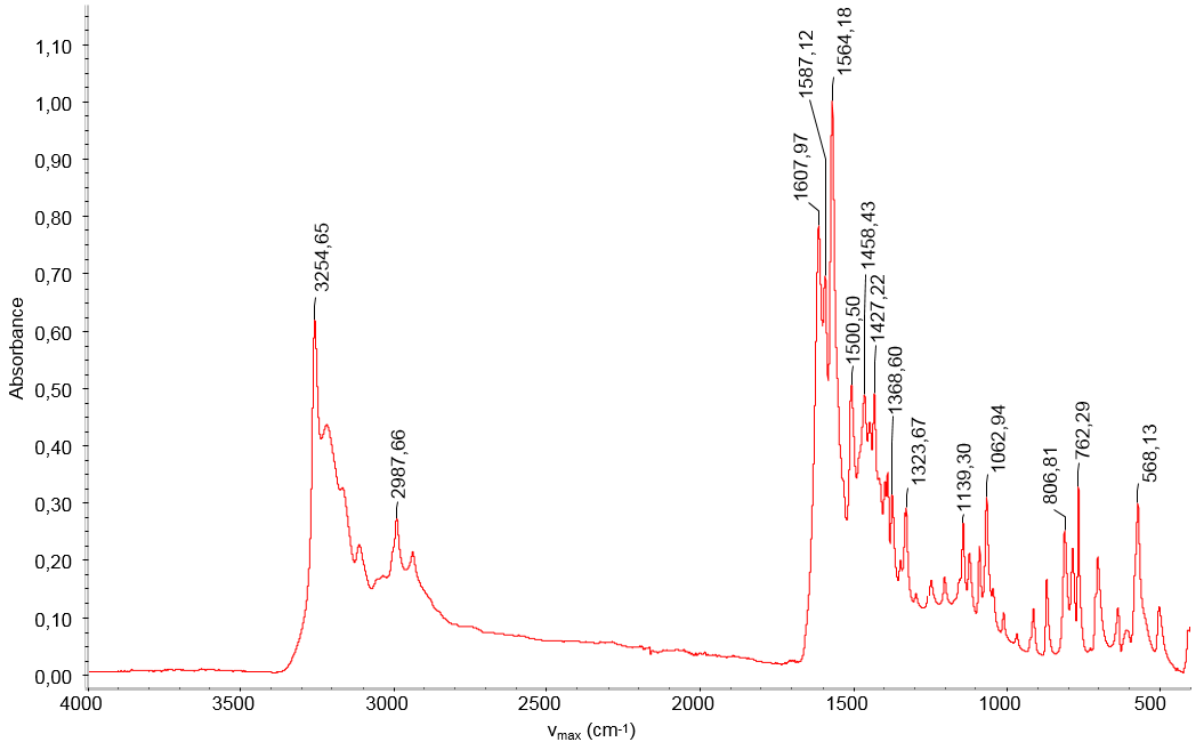

Figure S1. ATR-FT-IR (with ATR correction) of RM33.

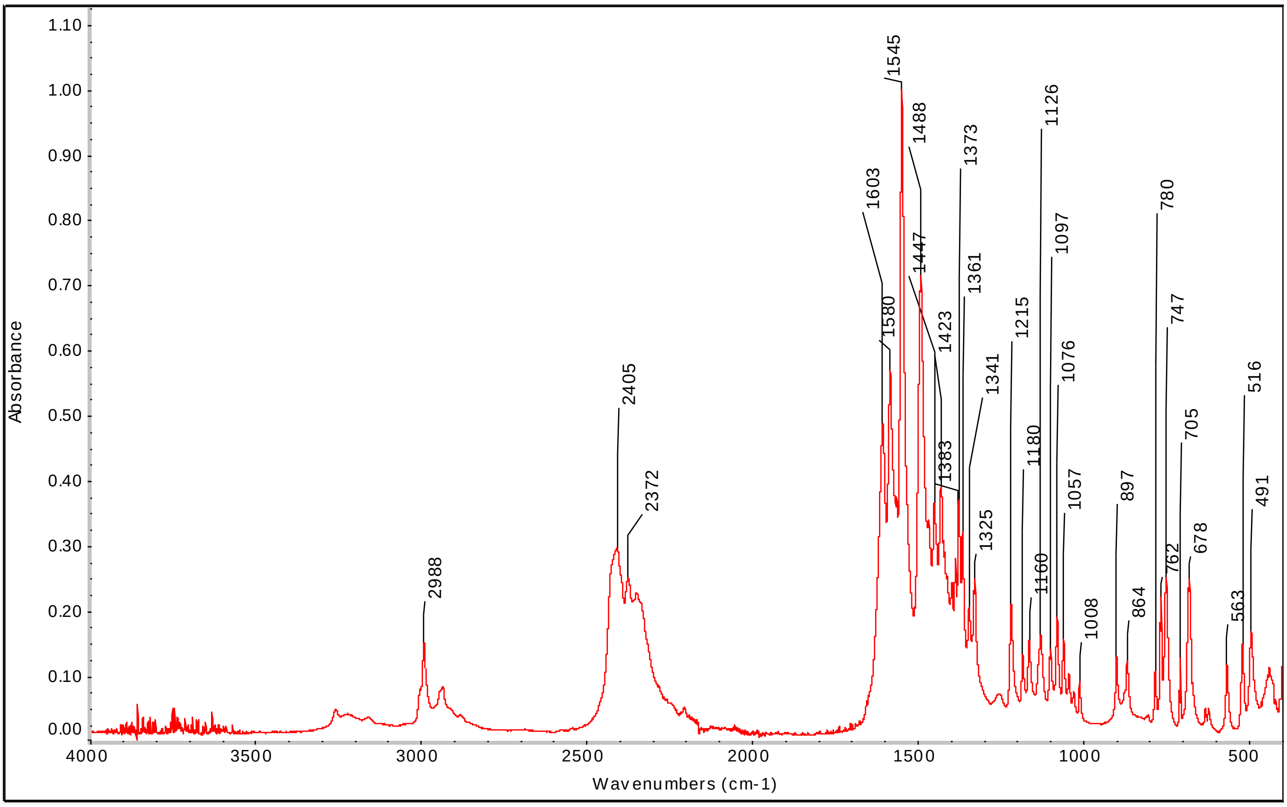

Figure S2. ATR-FT-IR (with ATR correction) spectrum of *N*-deuterated isotopologue of RM33.

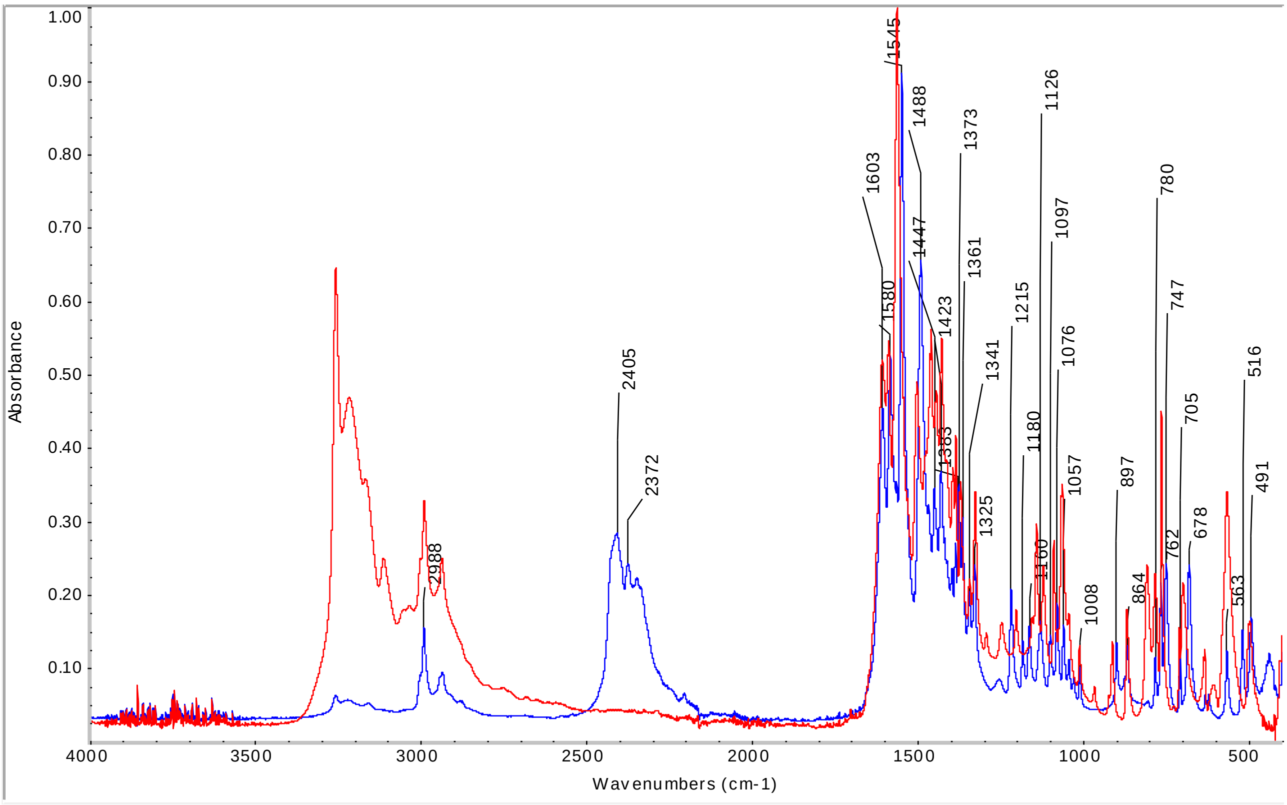

Figure S3. Superimposed ATR-FT-IR spectra of RM33 (red) and its *N*-deuterated isotopologue RM33D (blue)

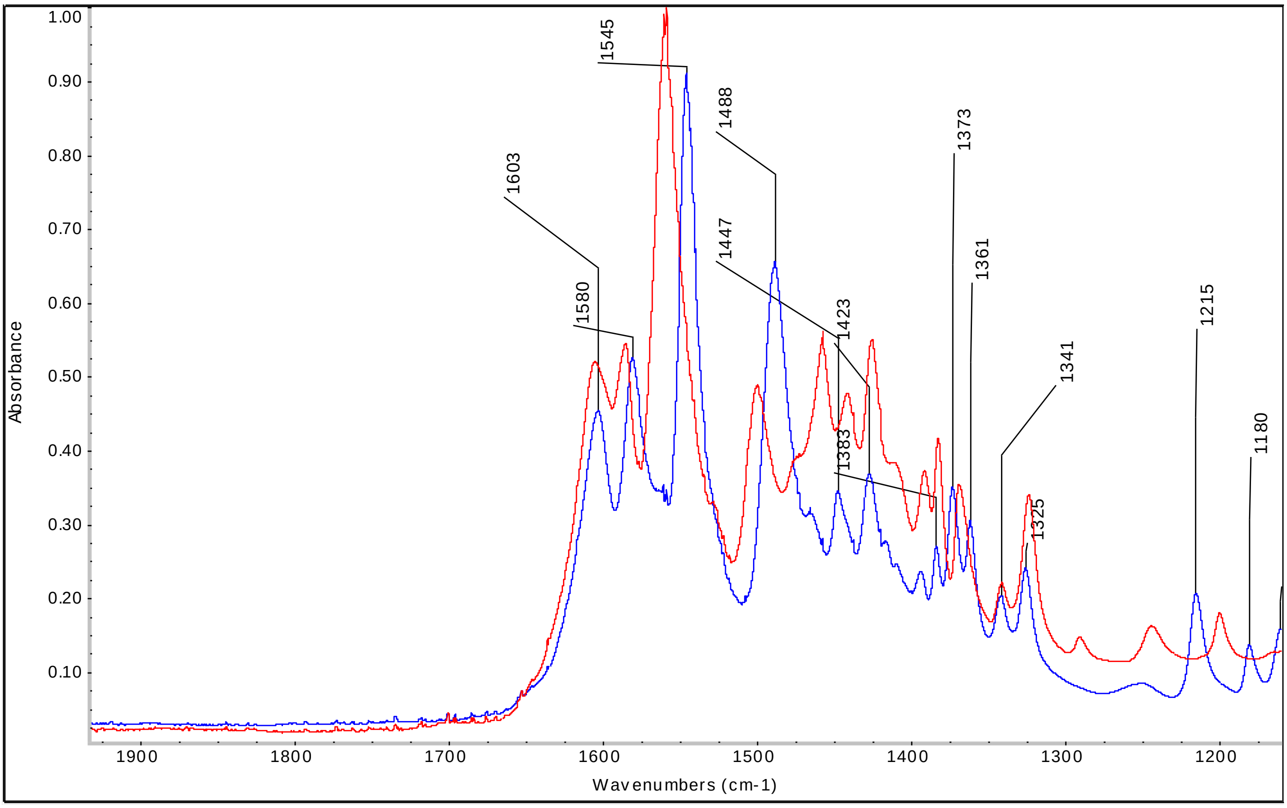

Figure S4. Superimposed ATR-FT-IR spectra (carbonyl region) of RM33 (red) and its N-deuterated isotopologue RM33D (blue).

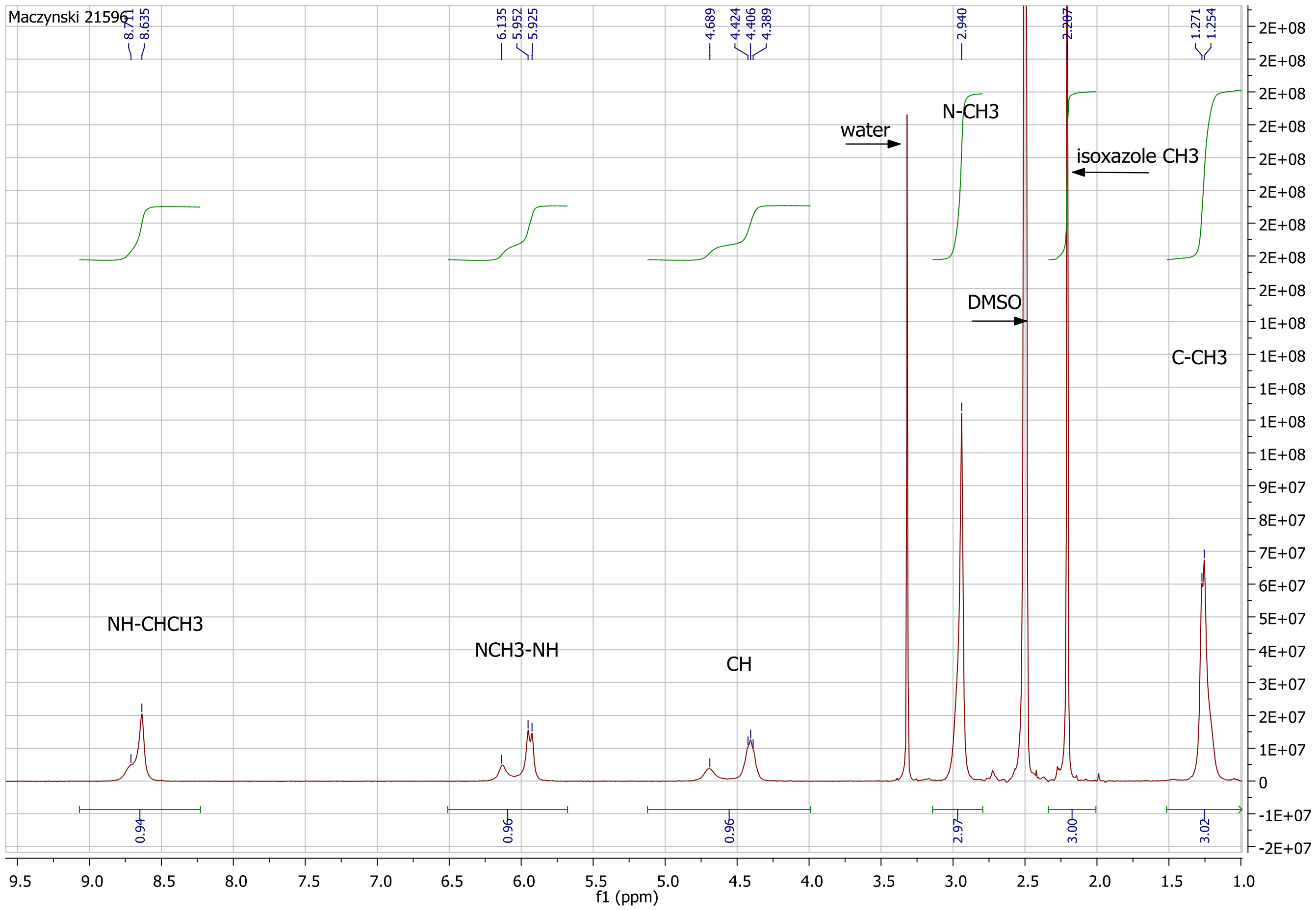

Figure S5. ^{1}H NMR (in DMSO-$_{d6}$) of RM33.

F2 - Acquisition Parameters for ^{1}H NMR (in DMSO-$_{d6}$) of RM33 shown above:

Date_ 20200702
Time 10.14
INSTRUM spect
PROBHD 5 mm BBI 1H/D-
PULPROG zg30
TD 65536
SOLVENT DMSO
NS 16
DS 0
SWH 6172.839 Hz
FIDRES 0.094190 Hz
AQ 5.3084660 sec
RG 80.6
DW 81.000 usec
DE 8.00 usec
TE 297.9 K
D1 1.00000000 sec
TD0 1
======== CHANNEL f1 ========
NUC1 1H
P1 10.40 usec
PL1 2.00 dB
SFO1 300.1518535 MHz

F2 - Processing parameters:

SI 32768
SF 300.1500045 MHz
WDW EM
SSB 0
LB 0.30 Hz
GB 0
PC 20.00

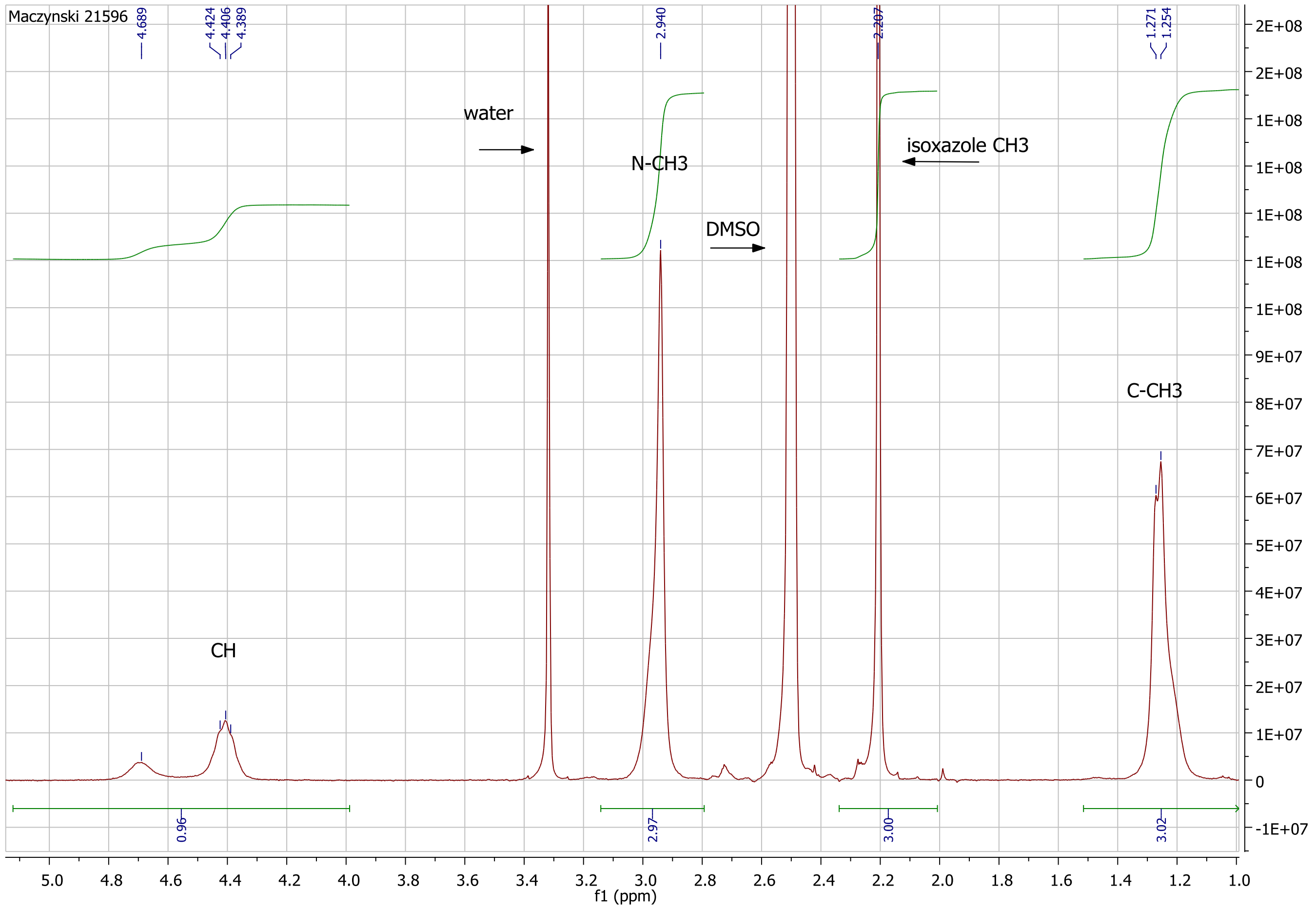

Figure S5A. ^{1}H NMR (in DMSO-d_6) of RM33. The spectrum is the same as in Figure S5, but the chemical shift range has been narrowed for the better visibility of signal splitting.

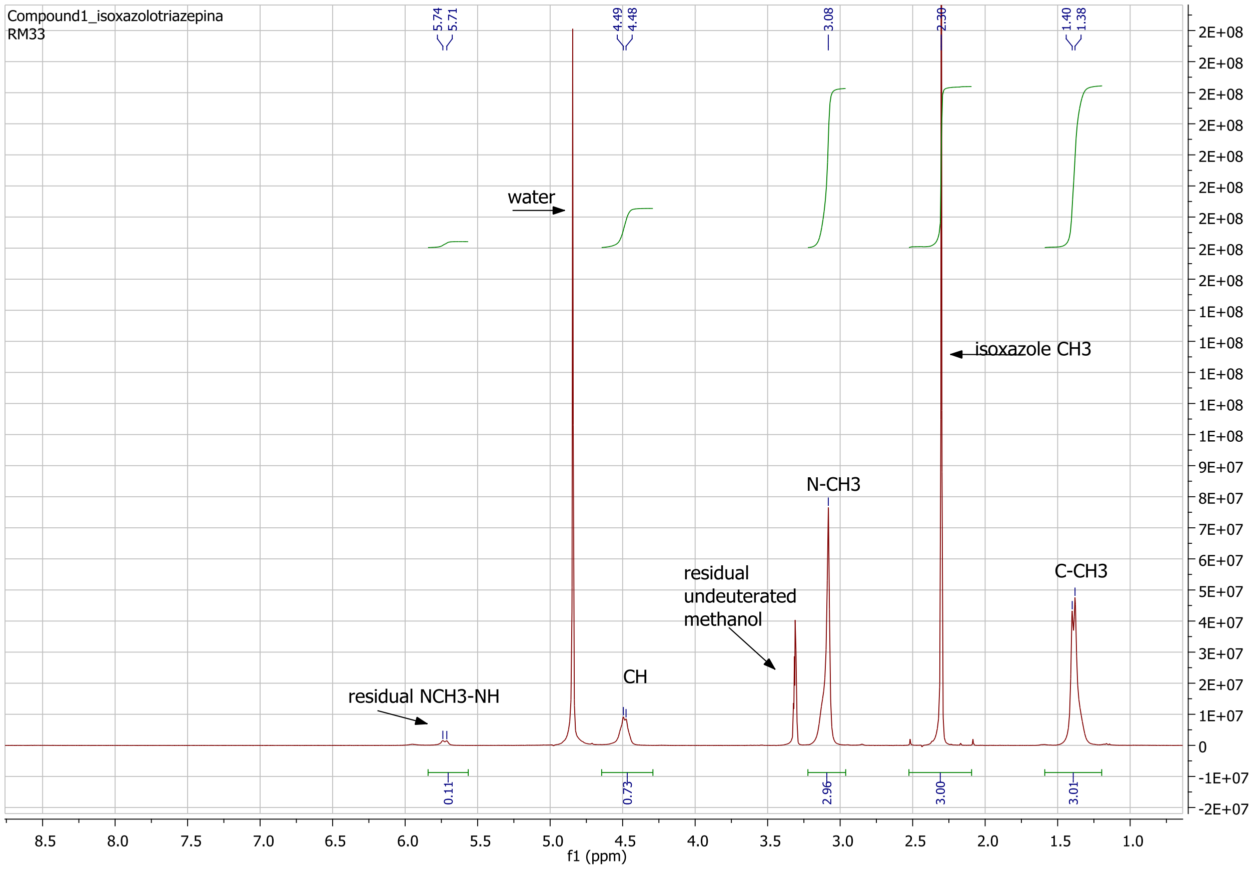

Figure S6. ^{1}H NMR (in CD_3OD) of RM33. Spectrum was performed after ten minutes after dissolvation of RM33 in CD_3OD

<u>F2 - Acquisition Parameters for ^{1}H NMR (in CD_3OD) of RM33 shown above</u>:
Date_ 20201002
Time 15.19
INSTRUM spect
PROBHD 5 mm BBI 1H/D-
PULPROG zg30
TD 65536
SOLVENT MeOD
NS 16
DS 0
SWH 6172.839 Hz
FIDRES 0.094190 Hz
AQ 5.3084159 sec
RG 71.8
DW 81.000 usec
DE 8.00 usec
TE 298.7 K
D1 1.00000000 sec
TD0 1
======== CHANNEL f1 ========
NUC1 1H
P1 10.40 usec
PL1 2.00 dB
SFO1 300.1518535 MHz
<u>F2 - Processing parameters</u>
SI 32768
SF 300.1500045 MHz
WDW EM
SSB 0
LB 0.30 Hz
GB 0
PC 20.00

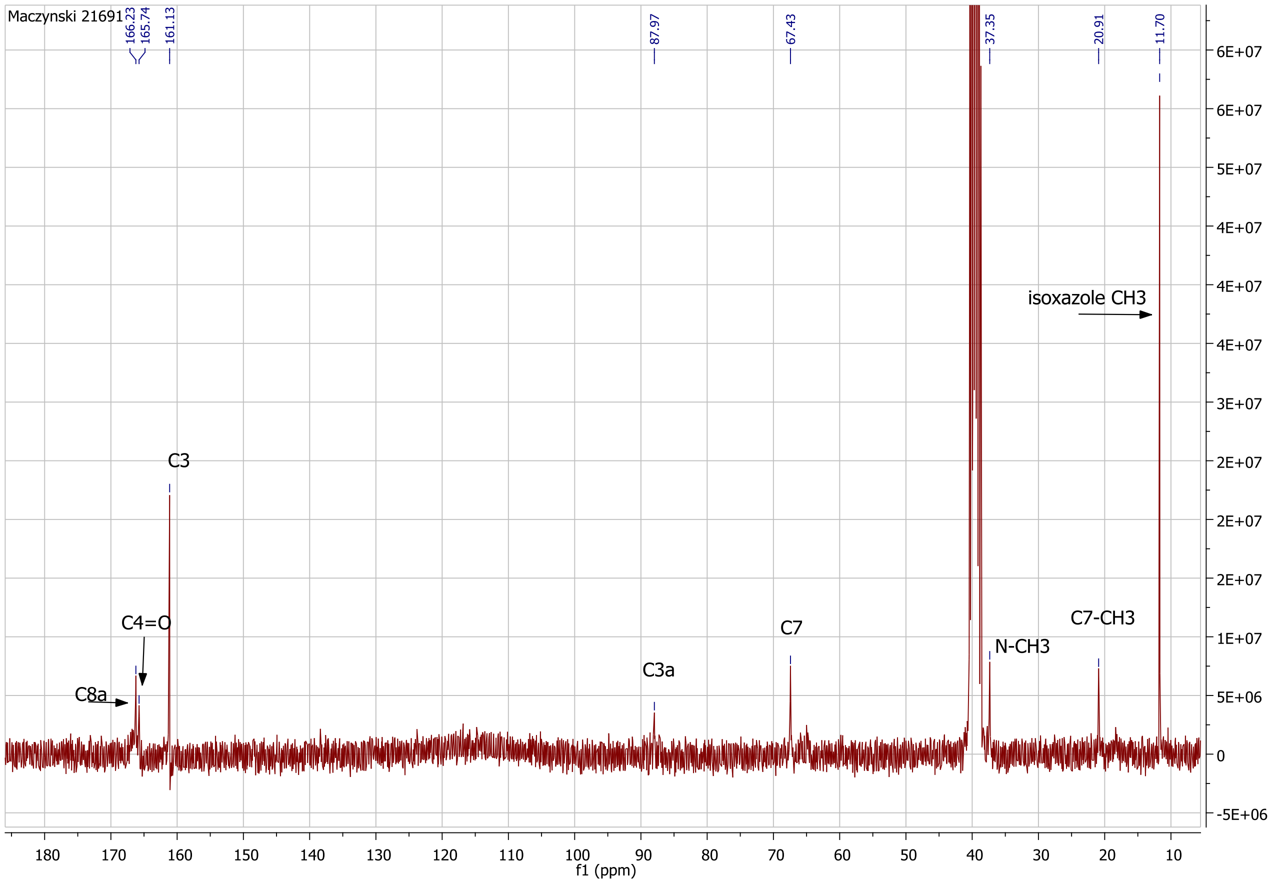

Figure S7. ^{13}C NMR (in DMSO-d_6) of RM33.

F2 - Acquisition Parameters for ^{13}C NMR (in DMSO-$_{d6}$) of RM33 shown above:
Date_ 20200921
Time 15.27
INSTRUM spect
PROBHD 5 mm BBI 1H/D-
PULPROG zgpg30
TD 65536
SOLVENT DMSO
NS 5120
DS 2
SWH 17985.611 Hz
FIDRES 0.274439 Hz
AQ 1.8219508 sec
RG 13004
DW 27.800 usec
DE 20.00 usec
TE 300.0 K
D1 2.00000000 sec
d11 0.03000000 sec
DELTA 1.89999998 sec
TD0 1
======== CHANNEL f1 ========
NUC1 13C
P1 11.00 usec
PL1 -6.00 dB
SFO1 75.4803248 MHz
======== CHANNEL f2 ========
CPDPRG2 waltz16
NUC2 1H
PCPD2 100.00 usec
PL2 2.00 dB
PL12 21.45 dB
PL13 23.00 dB
SFO2 300.1512006 MHz
F2 - Processing parameters:
SI 32768
SF 75.4728157 MHz
WDW EM
SSB 0
LB 1.00 Hz
GB 0
PC 2.00

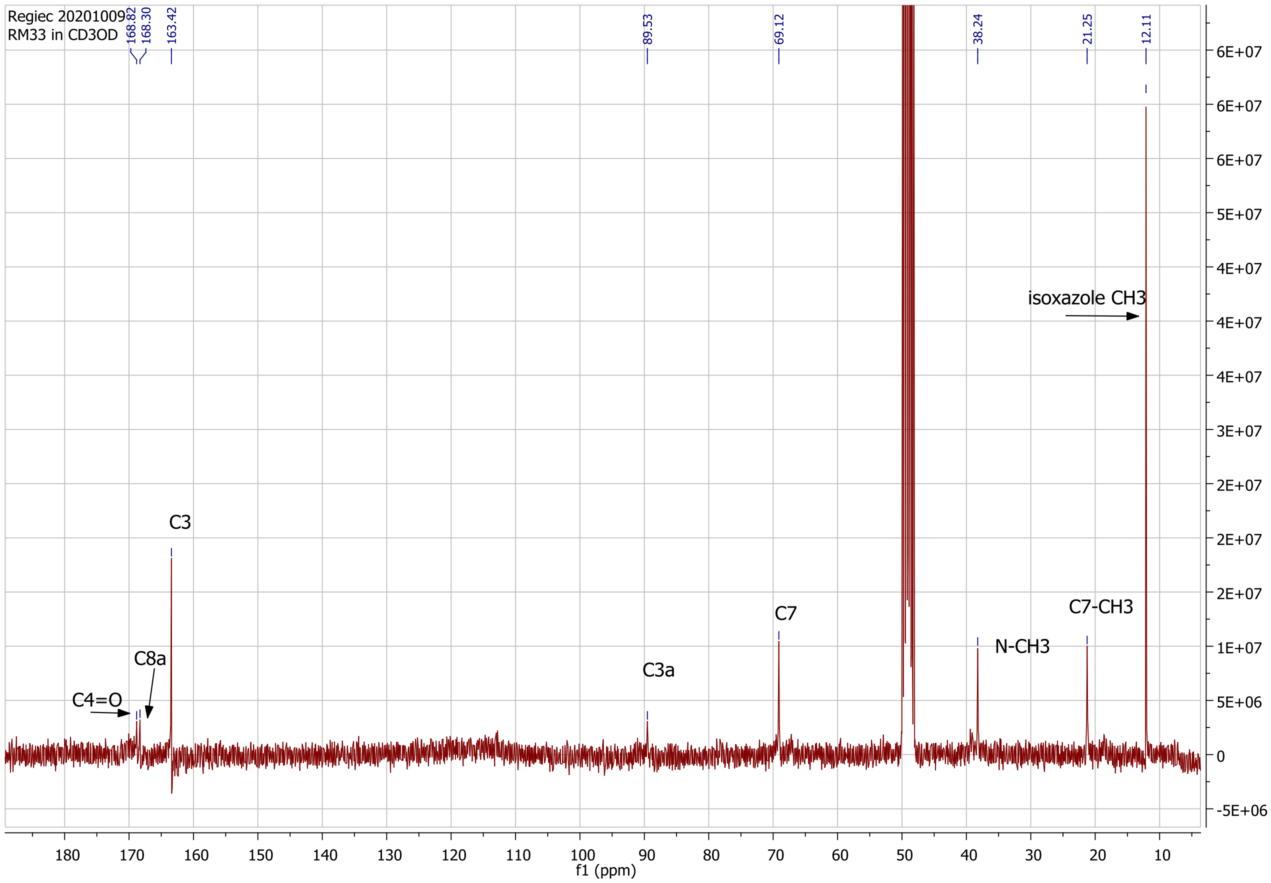

Figure S8. ^{13}C NMR (in CD_3OD) of RM33.

F2 - Acquisition Parameters for ^{13}C NMR (in CD_3OD) of RM33 shown above:
Date_ 20201009
Time 21.49
INSTRUM spect
PROBHD 5 mm BBI 1H/D-
PULPROG zgpg30
TD 65536
SOLVENT MeOD
NS 5120
DS 2
SWH 15060.241 Hz
FIDRES 0.229801 Hz
AQ 2.1758451 sec
RG 13004
DW 33.200 usec
DE 20.00 usec
TE 300.0 K
D1 2.00000000 sec
d11 0.03000000 sec
DELTA 1.89999998 sec
TD0 1
======== CHANNEL f1 ========
NUC1 13C
P1 11.00 usec
PL1 -6.00 dB
SFO1 75.4803248 MHz
======== CHANNEL f2 ========
CPDPRG2 waltz16
NUC2 1H
PCPD2 100.00 usec
PL2 2.00 dB
PL12 21.45 dB
PL13 23.00 dB
SFO2 300.1512006 MHz
F2 - Processing parameters
SI 32768
SF 75.4726708 MHz
WDW EM
SSB 0
LB 1.00 Hz
GB 0
PC 2.00

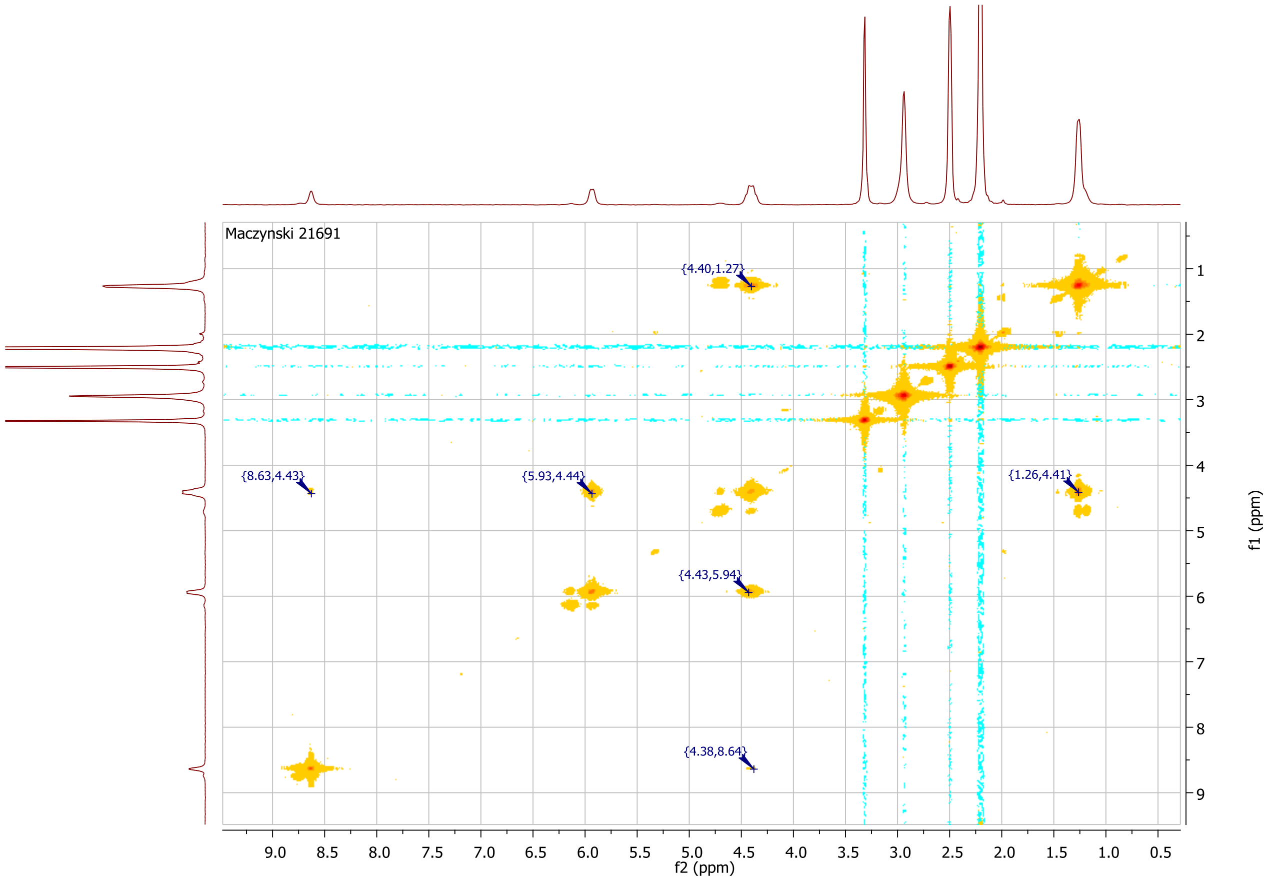

Figure S9A. ^{1}H-^{1}H 2D-COSY45 spectrum (in DMSO-*d6*) of RM33

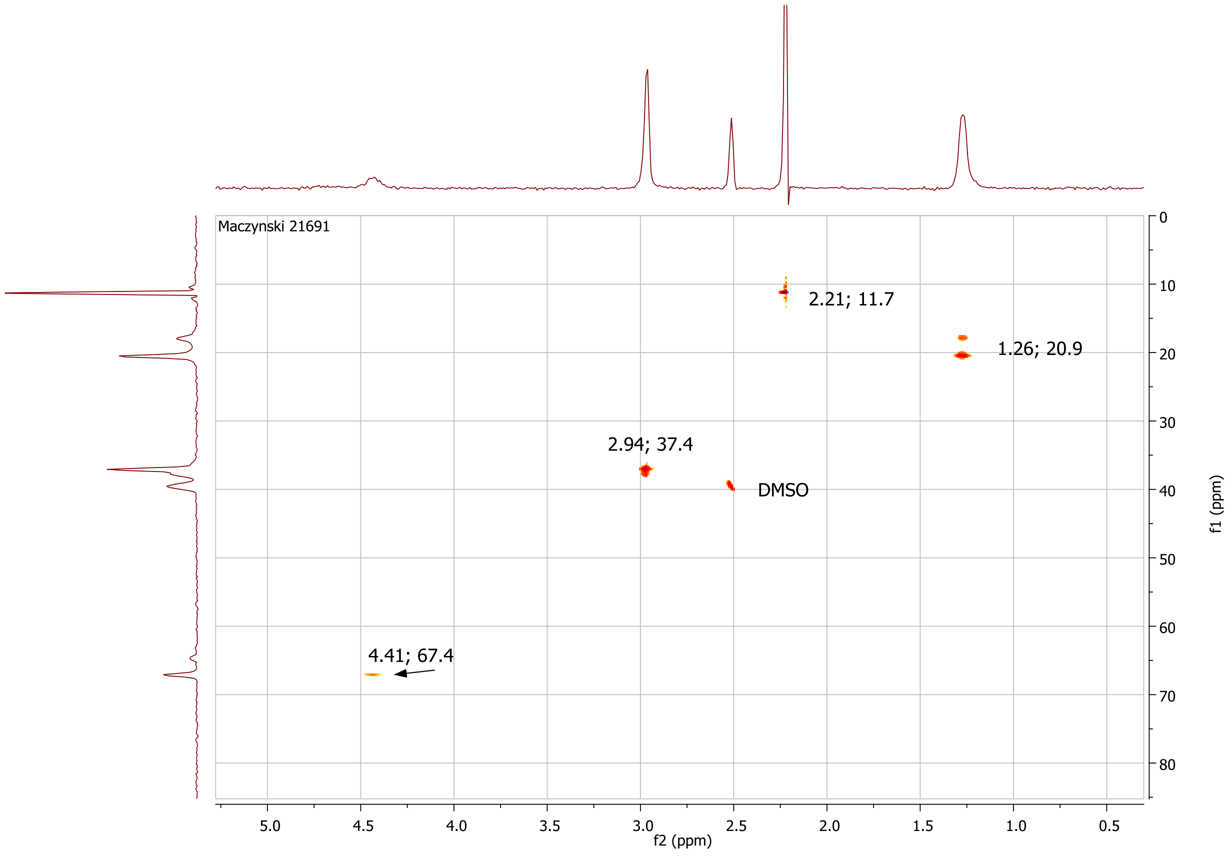

Figure S9B. ^{1}H-^{13}C 2D HSQC spectrum (in DMSO-*d6*) of RM33

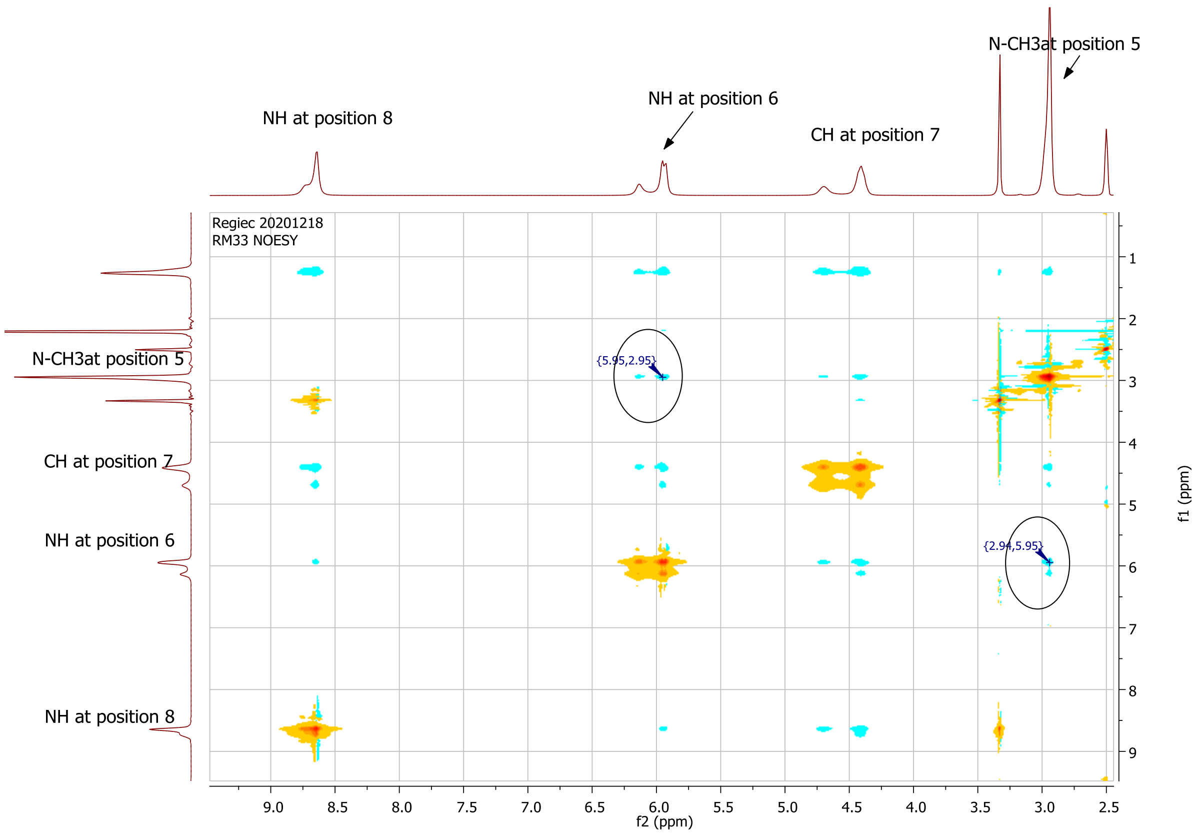

Figure S9C. NOESY spectrum (in DMSO-*d6*) of RM33

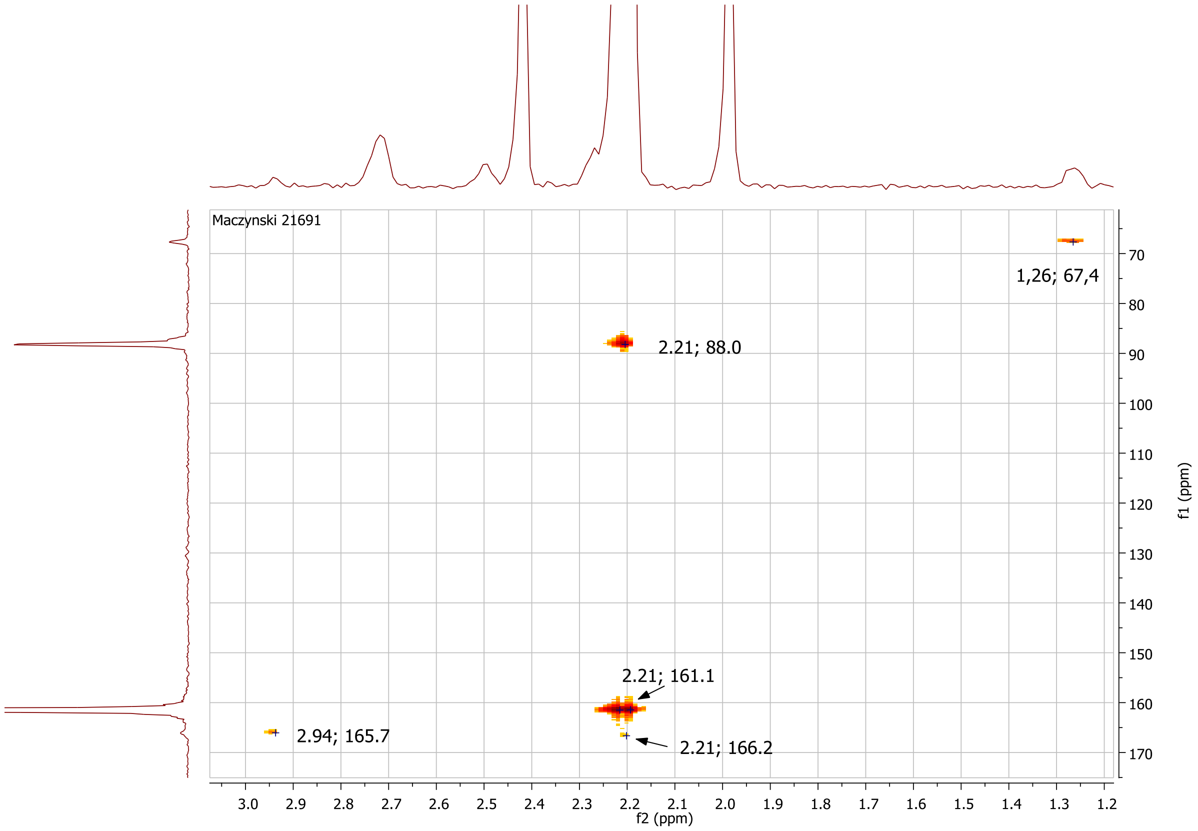

Figure S9D. 2D ^{1}H-^{13}C HMBC spectrum (in DMSO-*d6*) of RM33

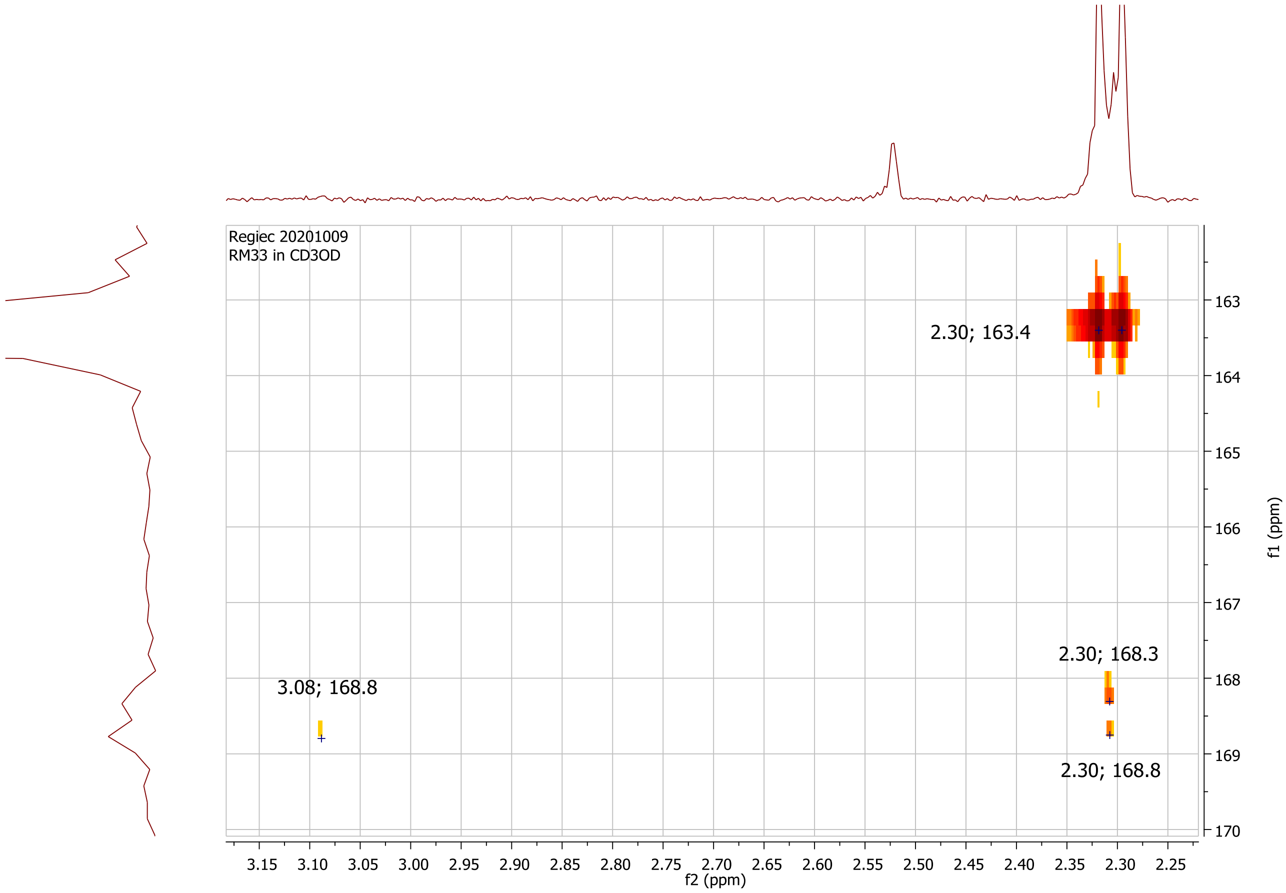

Figure S9E. 2D ^{1}H-^{13}C HMBC spectrum (in CD_3OD) of RM33

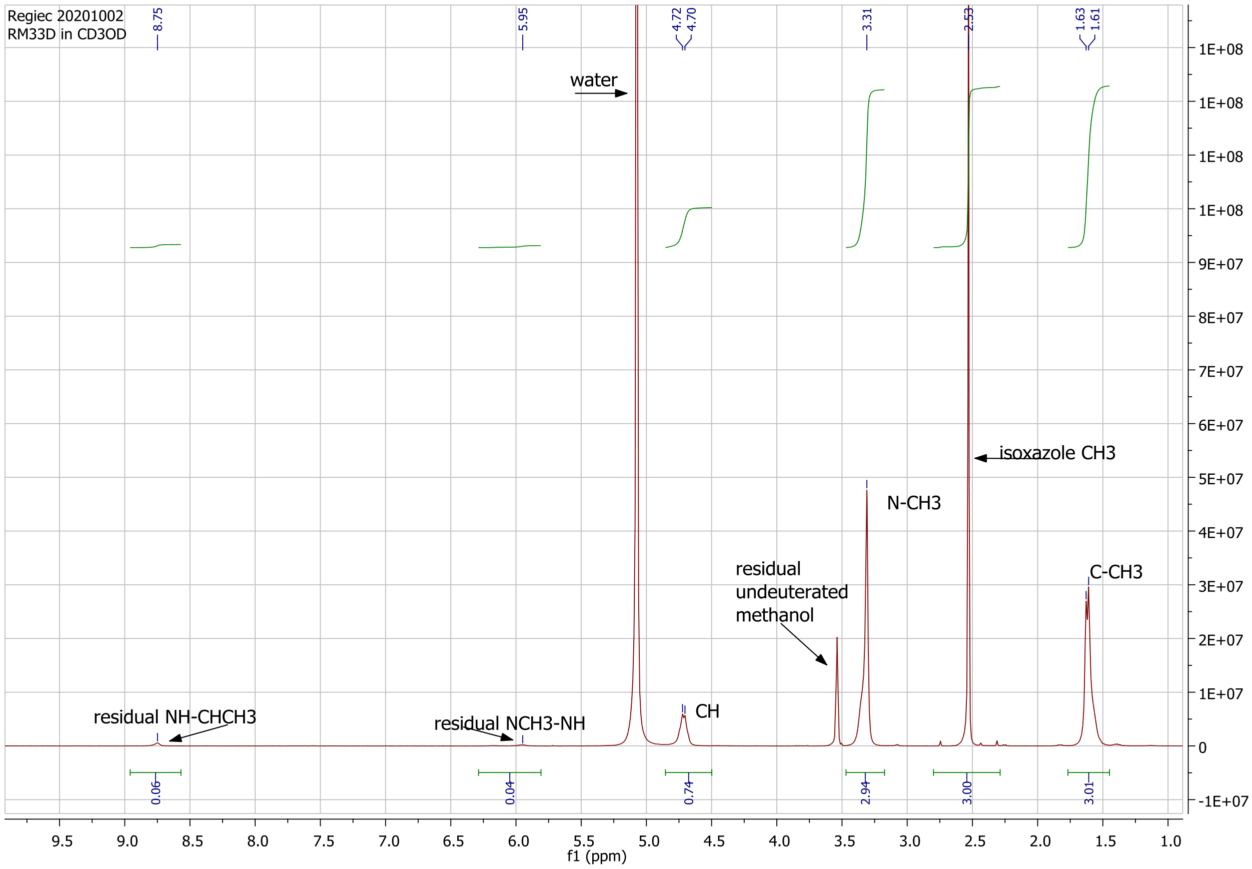

Figure S10. ^{1}H NMR (in CD_3OD) of *N*-deuterated isotopologue (RM33D) of RM33.

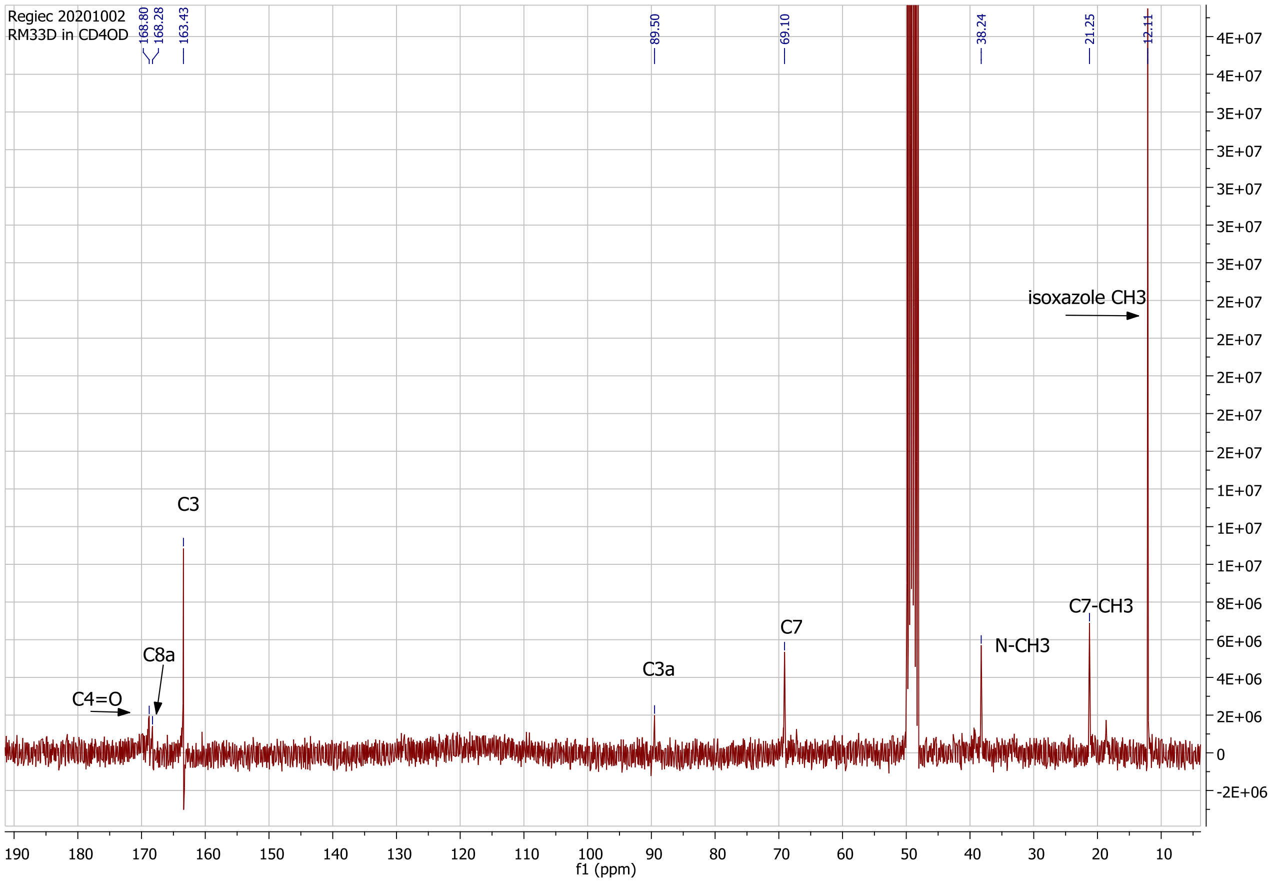

Figure S11. ^{13}C NMR (in CD_3OD) of *N*-deuterated isotopologue (RM33D) of RM33.

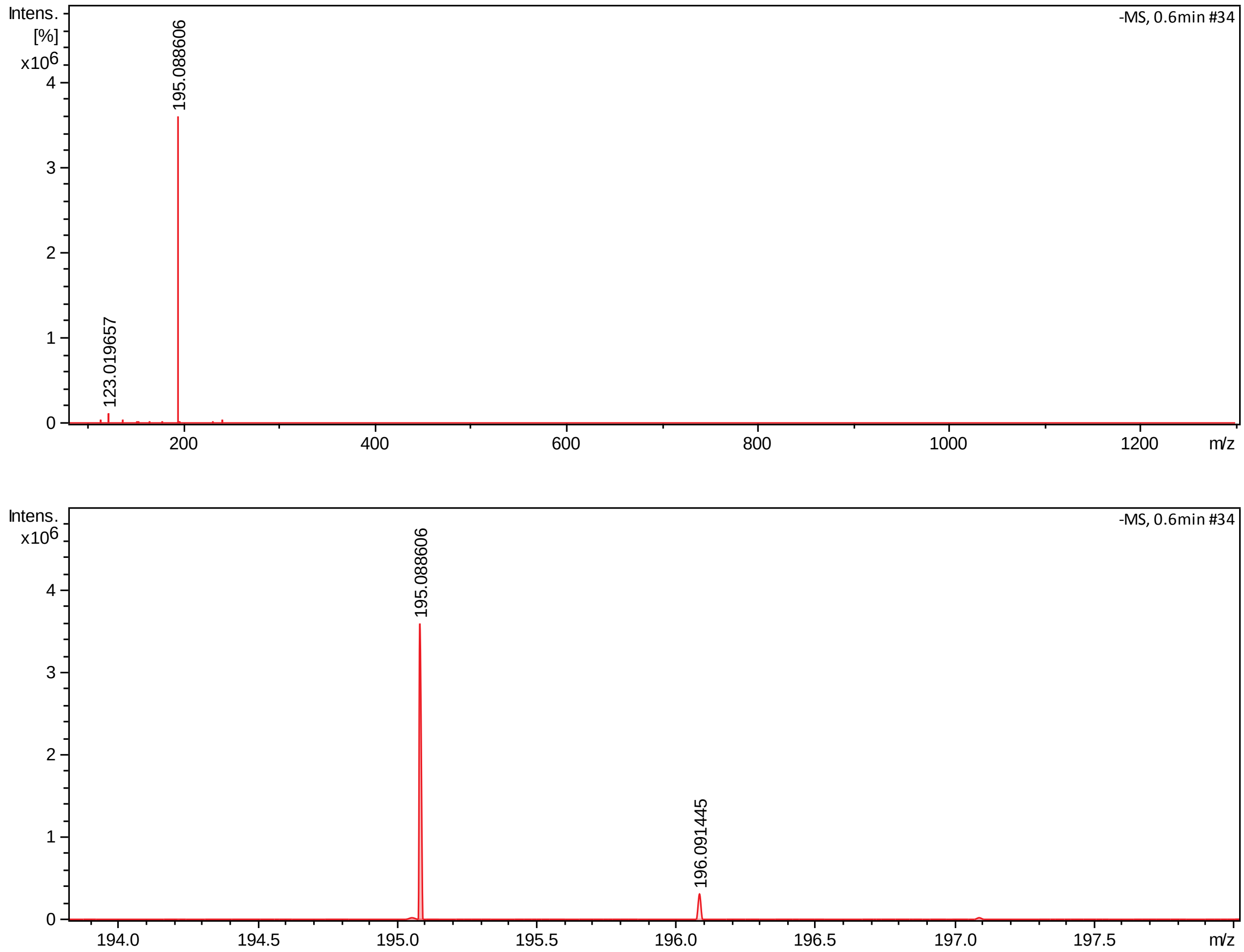

Figure S12. HR-ESI-MS (negative ionization in CH_3OH) of RM33, whole spectrum (top), magnification of quasi molecular ion $[M-H]^-$ peak region (below), next to quasi-molecular ion (100% relative intensity), two small isotope quasi-molecular ion peaks are visible, i.e. 196.091418 (8.50%) and 197.093193 (0.70% relative intensity).

2. Biology

2.1. Determination of RM33 toxicity by morphologic examination by microscopy

For evaluation of RM33 toxicity by microscopic parameters, A549 cell line were treated with a wide concentration range (0.78-200 µg/mL) of RM33 for 48h and monitored for changes in morphology. The representative photographs of the cultures, containing RM33 at concentration of 100 µg/mL and 200 µg/mL from a phase contrast microscope, are shown in Figure S12.

Figure S13. Phase contrast image of A549 cells treated with RM33 at 100 μg/mL and 200 μg/mL. Cell growth, morphology and viability were used as parameters to determine the cytotoxicity of the compound

A) A549 culture in the absence of RM33 – normal appearance of the cells

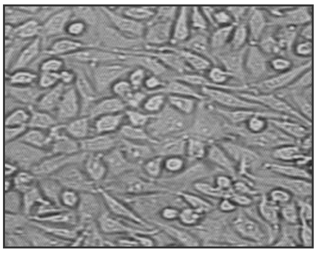

B) A549 cell culture with 1% phenol in the medium – a strong toxic effect

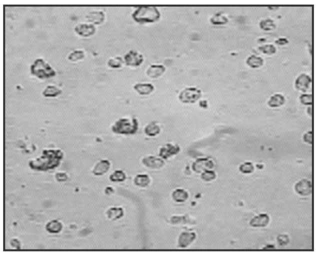

C) A549 cells cultured with RM33 at a concentration of 100 μg/mL – no toxic effect

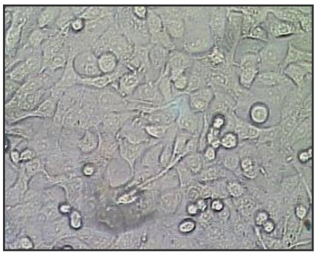

D) A549 cells cultured with RM33 at a concentration of 200 μg/mL – a small toxic effect

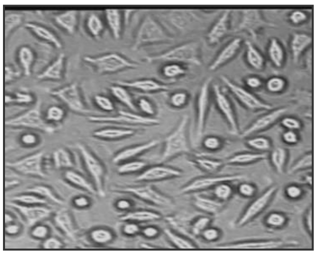

After 48h of culture with RM33 at a concentration of 200 μg/mL, a small cytotoxic effect of the compound (13% of dead cells) was additionally determined by MTT method. The morphology of the cell culture at this concentration (Figure S12D) allowed to classify RM33 as weakly toxic according to the 4-grade scale – Criteria of toxicity effect based on changes in cell morphology (EN ISO 10993-5:2009. Biological evaluation. Part 5: Test for in vitro cytotoxicity. International Organization for Standardization, Geneva, Switzerland, 2009 [3]). RM33 at 100 μg/mL had no toxic effect on morphology of A549 cells (Figure S12C).

2.2. Effects of RM33 on viability of mouse lymphocytes and proliferation of mouse and human lymphocytes to mitogens

Cells from mouse spleens, thymuses and bone marrow were incubated overnight with RM33 (20 μg/mL). The cell viability was measured by MTT colorimetric method. In Table S1 the effects of the compound as compared to appropriate DMSO control are presented. RM33 had a small (18.5%) negative effect of the viability of splenocytes and no effect on thymocytes. On the other hand, the compound and an enhanced the viability of bone marrow cells.

Table S1. The viability of cells from lymphatic organs measured by the MTT colorimetric method

Organ	% viable cells
Spleen	81.5
Thymus	101.8
Bone marrow	117.8

The effect of RM33, at 1.25-5 μg/mL concentration range, on the proliferative response of mouse splenocytes to ConA is presented in Table S2. CsA was used a reference compound. No significant effect of RM33 on the proliferative response of splenocytes was found. A small stimulatory tendency was registered at 2.5 and 5 μg/mL concentration.

Table S2. Effect of RM33 on ConA-induced mouse splenocyte proliferation

Experimental group	(μg/mL)	OD 550/630 nm	±SE
-	-	0.056	0.001
ConA control	-	0.734	0.005
RM33	1.25	0.628	0.010
	2.5	0.594	0.014
	5	0.506	0.001
Solvent*	1.25	0.626	0.015
	2.5	0.564	0.014
	5	0.477	0.010
CsA	1.25	0.038*	0.002
	2.5	0.025*	0.001
	5	0.014*	0.000

*0.64 : 0.36/ ethanol : cremophor

Likewise, RM33 at 0.1-5 μg/mL concentration range had no regulatory effect on the PHA-induced proliferation of human PBMC (Table S3).

Table S3. Effect of RM33 on PHA-induced human PBMC proliferation, *versus control, $p<0.05$

Experimental group	(μg/mL)	OD 550/630 nm	±SE
-	-	0.047	0.002
PHA control	-	0.253	0.004
DMSO	0.1	0.239	0.003
	1	0.241	0.007
	5	0.255	0.008
RM33	0.1	0.235	0.004
	1	0.250	0.007
	5	0.260	0.007
CsA	0.1	0.104*	0.002
	1	0.086*	0.001
	5	0.074*	0.002

2.3. Effect of RM3 on the secondary humoral immune response *in vitro* to SRBC

RM33, added to cell cultures of splenocytes derived from SRBC-immunized mice, had no effect on development of secondary immune response *in vitro* (Table S4). CsA had a profound inhibitory action on the number of AFC.

Table S4. Effect of RM33 on the secondary humoral immune response to SRBC *in vitro,* *versus DMSO control, $p<0.05$

Experimental group	μg/mL	AFC/10^6	±SE
Background	-	423	30.81
Control	-	10028	123.20
DMSO	0.1	9847	183.60
	1	9889	60.09
	5	10055	55.56
RM33	0.1	9597	127.29
	1	10055	242.41
	5	9847	149.71
CsA	0.1	2229*	79.96
	1	750*	35.80
	5	208*	17.90

2.4. Effects of RM33 on expression of signaling molecules in cell lines corresponding to immature B cells and granulocyte/monocyte precursors

The results regarding effects of RM33 on the immature B cell line WEHI 231 did not reveal significant changes in expression of signaling molecules associated with apoptosis or cell differentiation or activation (Tables S5 and S6).

Table S5. Effect of RM33 on expression of signaling molecules in WEHI 231 cells

Casp-3	Casp-8	Casp-9	NFκB1	Bcl-2	Fas
0.35	0.98	0.99	0.84	1.09	0.78

Table S6. Effect of RM33 on expression of MAP kinases in WEHI 231 cells

ERK-1	ERK-2	p38a	p38b	p38g	p39d	JNK
0.93	0.90	0.96	1.29	0	0.38	1.34

Likewise, in the case of promyelocytic HL-60 cell line no significant changes in expression of signaling molecules were found (Table S7). The increases in expression of ERK-1/ERK-2 and caspase 8 might be of interest in terms of potential promotion of cell differentiation by RM33.

Table S7. Effect of RM33 on expression of signaling molecules in promyelocytic HL-60 cell line

ERK-1	ERK-2	JNK	p38-α	p38-β	p38-γ	p38-δ	Casp-3	Casp-7	Casp-8	Casp-9	BCl-2	Fas	NFκB1	P53
2.3	5.2	1.3	1.7	1.5	1.1	0	1.3	0.9	1.9	1.3	0	0	0.9	0

2.5. Effects of RM33 on metabolism and pinocytic activity of J774 macrophage cell line

RM33 compound, at a concentration range 1.5-50 μg/mL, had also a minor inhibitory effect on metabolism of J774 cells (Table S8).

Table S8. Effect of RM33 (at indicated concentrations) on metabolism of J774 macrophage cell line, *versus DMSO control, p<0.05

Experimental group	μg/mL	OD 550/630 nm	% inhibition
DMSO control		0.845	
		0.841	
		0.833	
		0.818	
		0.816	
		0.793	
RM33	1.562	0.849	0.0
	3.125	0.813	3.3
	6.25	0.764	8.3
	12.5	0.745	8.9
	25	0.756	7.4
	50	0.740	6.7

The effects of RM33 at a concentration range 25-100 μg/mL, on the endocytic activity of macrophage J774 cell line revealed a regulatory characteristics, i.e. 25 μg/mL concentration was stimulatory and 100 μg/mL was inhibitory (Figure S13).

Figure S14. Determination of endocytic activity of J774 cell line incubated with RM33 at indicated concentrations

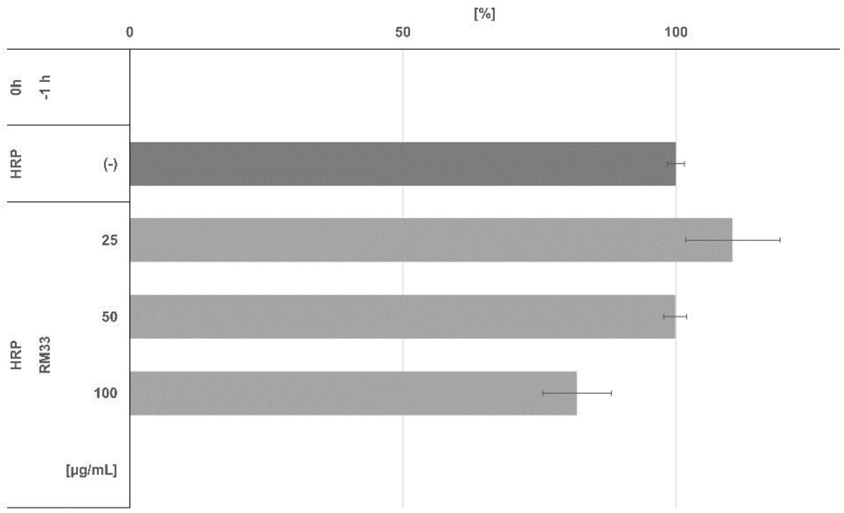

2.6. Effect of RM33 on IL-8 production by human blood cells

Table S9 combines results from four experiments with human whole blood cell cultures treated with LPS and assayed for IL-8 production. It appeared that RM33 at concentration of 25 μg/mL weakly inhibited IL-8 production.

Table S9. Effect of RM33 on LPS-induced IL-8 production in human whole blood cell cultures; *versus DMSO control, $p < 0.05$

RM33 concentration (μg/mL)	% inhibition	±SE
10	2.8	2.5
25	4.9*	1.2

2.7. The sequences of primers used in the study

The sequences of primers for respective signaling molecules, both for human and mouse cells, are presented in Table S10.

Table S10. The sequences of primers

Gene	Vector	Sequence [5' - 3'] Human	Sequence [5' - 3'] Mouse
GAPDH	F<br>R	AGTCAGCCGCATCTTCTTTT<br>TGAGGTCAATGAAGGGGTCA	CTCCACTCACGGCAAATTCAACGG<br>GGTGAAGACACCAGTAGACTCCA
ERK1	F<br>R	GTGGCCCCAGTTCAATCTC<br>GGGTTTGAATGAGATGAGGG	TACCTGGACCAGCTCAACCACATT<br>AGCAGGTCAAGAGCTTTGGAGTCA
ERK2	F<br>R	TATTACGACCCGAGTGACGA<br>AAGAACACCGATGTCTGAGC	GGAACAGGTTGTTCCCAAATGCTGAC<br>CTCCTTAGGTAAGTCGTCCAACTCC
p38α	F<br>R	CTGAGGTATATCCACGCTGC<br>TGTAGCGCATCCAATTCAAGA	GAAGTCATCAGCTTTGTGCCACC<br>CCACAAAGATAGGTGGACAGACG
p38β	F<br>R	ACAGTGGATATCTGGTCCGT<br>ATATATGTCCGGGCGTGTTC	TGGCACCCATGAAATTGAGCAGTG<br>GGCATATGTACACATCCGTGCATTCC
p38γ	F<br>R	TATGCGTCTGACAGGAACAC<br>GGGCCGCTGTAATTCTCTTA	GGACATTTGGTCCGTTGGCTGCAT<br>CCATGTAGTTCTTGGCCTCTGC
p38δ	F<br>R	GGCACATGGCCTGTGTAATA<br>TAGGAAATGTCCCCCACCTT	AGAAGGTGGCCATCAAGAAGCTGA<br>TCCAGAAGCCCAATGACGTTCTCA
JNK	F<br>R	GCTCTGCGTCACCCATACAT<br>GCATCTGTGCTGAAGGCTGA	AGCTCGGAACACCTTGTCCTGAAT<br>GGAGAGCTTCATCTACGGAGATCC
Casp-3	F<br>R	TTCAGAGGGGATCGTTGTAGAAGTC<br>CAAGCTTGTCGGCATACTGTTTCAG	TGTCATCTCGCTCTGGTACG<br>AAATGACCCCTTCATCACCA
Casp-7	F<br>R	AGTGACAGGTATGGGCGTTC<br>CGGCATTTGTATGGTCCTCT	
Casp-8	F<br>R	GCAAAAGCACGGGAGAAAG<br>GGATACAGCAGATGAAGCAG	ACAATGCCCAGATTTCTCCCTAC<br>CAGACAGTATCCCCGAGGTTTG
Casp-9	F<br>R	GAGTCAGGCTCTTCCTTTG<br>CCTCAAACTCTCAAGAGCAC	AGCCAGATGCTGTCCCATAC<br>CAGGAGACAAAACCTGGGAA
Bcl-2	F<br>R	TATAAGCTGTCGCAGAGGGGCTA<br>GTACTCAGTCATCCACAGGGCGAT	CTCGTCGCTACCGTCGTGACTTCG<br>CAGATGCCGGTTCAGGTACTCAGTC
Fas	F<br>R	GACAAAGCCCATTTTTCTTCC<br>ATTTATTGCCACTGTTTCAGG	GCGATGAAGAGCATGGTTTAG<br>GGCTCAAGGGTTCCATGTT
NFκB	F<br>R	GAGTTACCTACCAGGGCTATTC<br>CTCTCCTCATCCTCACTCTCT	GAAATTCCTGATCCAGACAAAAAC<br>ATCACTTCAATGGCCTCTGTGTAG
p53	F<br>R	AGATAGCGATGGTCTGGC<br>TTGGGCAGTGCTCGCTTAGT	CCCGAGTATCTGGAAGACAG<br>ATAGGTCGGCGGTTCAT

3. Materials and Methods

3.1. Preparation of RM33 solutions for *in vitro* studies

The compound was initially dissolved in dimethylsulfoxide (DMSO) or in a mixture of ethanol/cremophor® (64/36 vol:vol ratio), by adding 1 mg of RM33 to 0.1 mL of the solvents. Next, a stock solution was prepared by dilution with the culture medium to attain the tested concentration. Control solutions of the solvents in the culture medium were also prepared and used in appropriate dilutions corresponding to respective concentrations of RM33 in cell cultures.

3.2. Determination of RM33 toxicity

A549 cell line (epithelial lung cancer cells) was treated with a wide concentration range (0.78-200 µg/mL) of RM33 for 48h and monitored for changes in morphology by light microscopy. Additionally the cell viability was determined by MTT colorimetric assay.

3.3. Isolation of human peripheral blood mononuclear cells PBMC

Venous blood from a single donor was withdrawn into heparinized syringes and diluted twice with phosphate-buffered saline (PBS). Peripheral blood mononuclear cells (PBMC) were isolated by centrifugation on Ficoll-uropoline gradient (density 1.077 g/mL) and centrifuged at 800×g for 20 min at 4°C. The interphase cells were then washed three times with Hanks' medium and re-suspended in a standard culture medium consisting of RPMI-1640 medium supplemented with 10% fetal calf serum (FCS), L-glutamine, 2-mercaptoethanol, sodium pyruvate and antibiotics (referred bellow to as the culture medium) at density of 2×10^6 cells/mL.

3.4. Proliferation of human PBMC to phytohemagglutinin (PHA)

Isolated PBMC were re-suspended in the culture medium and distributed into 96-well flat-bottom plates in 100 µL aliquots (2×105 cells/well). PHA was added at a concentration of 5 µg/mL. Cyclosporin A (CsA) was used a reference compound. The compounds were tested at a concentration range 0.1-5 µg/mL. DMSO at appropriate dilutions served as control. After a four-day incubation in a cell culture incubator, the proliferative response of the cells was determined by the colorimetric MTT method. The data are presented as a mean optical density (OD) at 550/650 nm ± standard error (SE) from quadruplicate determinations.

3.5. Proliferation of mouse splenocytes to concanavalin A (ConA)

The spleens were pressed against a plastic screen into 0.83% NH_4Cl solution to lyze erythrocytes (5 min incubation at room temperature). The cells were then washed twice with Hanks' medium, passed through a glass wool column to remove debris, and re-suspended in the standard culture medium (RPMI-1640, supplemented with 10% FCS, L-glutamine, sodium pyruvate, 2-mecraptoethanol and antibiotics). The cells were then distributed into 96-well flat-bottom tissue culture plates (Nunc) at a density of 2×10^5/well. 2.5 µg/mL ConA was added to induce cell proliferation. CsA was used a reference compound. The compounds were tested at a concentration range 1.25-5 µg/mL. DMSO at appropriate dilutions served as control. After a 3-day incubation, the cell proliferation was determined using the colorimetric MTT assay according to Hansen et al. [1]. The results are presented as the mean OD at 550/630 nm ±SE from quadruplicate determinations.

3.6. Colorimetric MTT assay for cell growth and kill

Briefly, 25 µL of MTT (5 mg/mL) stock solution was added per well at the end of cell incubation and the plates were incubated for 3 h in a cell culture incubator. Then, 100 µL of the extraction buffer (20% SDS with 50% DMF, pH 4.7) was added. After additional overnight incubation, the OD was measured at 550/630 nm wavelength (Dynatech).

3.7. Secondary immune response to SRBC *in vitro*

Mice were sensitized intraperitoneally with 0.2 mL of 5% sheep red blood cells (SRBC) suspension. After four days spleens from these mice were isolated, splenocyte single cell suspension prepared and suspended in the culture medium at a density of 5×10^6/mL. The cells were distributed to 24-well culture plates in 1 mL aliquots and 0.05 mL of 0.005% SRBC was added as antigen. CsA was used a reference compound. The compounds were added to the cultures in the beginning of the four-day incubation period at concentration range 0.1-5 µg/mL. The number of antibody-forming cells (AFC) in the cultures were determined using a method of local hemolysis in agar gel according to Mishell and Dutton [2] and the results are shown as mean values ± SE from from four independent experiments.

3.8. Effect on metabolism and endocytosis activity of J774 macrophage cell line

For determination of the effect of RM33 on metabolism of J774 macrophage cell line cells at a density of $2.5\text{x}10^4$/well was incubated for 24h with RM33 at 1.5-50 µg/mL concentrations. The cell viability was determined by the MTT colorimetric method and the results are shown as mean OD values from triplicate determinations as well as % inhibition vs DMSO control.
For determination of the endocytic activity of RM33 cells was plated at a density of 2.5×10^4/cells in a 96-well plate and left to adhere overnight (RPMI-1640 media supplemented with 1% FCS and antibiotics). On a next day the cells were pretreated with RM33 (or DMSO) for 1h prior to incubation with horseradish peroxidase (HRP, 25 µg/mL). After 2h incubation at 37°C in 5% CO_2, the cell monolayers were washed twelve times in PBS and lyzed in 0.05% (vol/vol) Triton X-100 in PBS. Aliquots of the cell lyzate were assayed for bound HRP by an enzymatic method, where 3,3',5,5'-tetramethylbenzidine is a substrate, and OD read at 650 nm wavelength. The results are shown as % inhibition vs DMSO control.

3.9. Induction and determination of IL-8 in human whole blood culture

The venous blood was taken from human donors to heparinized tubes. The blood was diluted 1:10 with RPMI-1640 medium with addition of antibiotics and distributed to 48-well plates. LPS from *E. coli* O111:B4 (100 ng/mL) was used for induction of cytokine. RM33 was used at concentration of 10 and 25 µg/mL. DMSO solution (0.25%) in the culture medium was used as in control culture. After an overnight incubation IL-8 level was determined in the supernatants (ELISA kit, BD cat. #555244). The results are shown as mean values ±SE from four independent experiments (four blood donors) as % inhibition versus DMSO control.

3.10. Statistics

Each experimental group consisted of 4 wells (determinations) for *in vitro* tests. The results were subjected to statistical analysis using analysis of variance (one-way ANOVA) in STATISTICA 7 for Windows. Brown-Forsyth's test was used to determine the homogeneity of variance between groups. Due to non-constant variance, the data were analyzed using the non-parametric the Kruskal-Wallis' analysis of variance, followed by Dunn's test to estimate the significance of the difference between groups. Significance was determined at $p < 0.05$. The results are presented as mean values ± SE.

References

[1]. Hansen, M. B.; Nielsen, S.E.; Berg, K. Reexamination and further development of a precise and rapid dye method for measuring cell growth/cell kill. *J. Immunol. Methods* **1989**, *119*, 203-210.

[2]. Mishell, R.I; Dutton, R.W. Immunization of dissociated spleen cell cultures from normal mice. *J Exp Med*, **1967**, 126, 423-442

[3]. PN-EN ISO 10993–12:2009 Biological evaluation of medical devices. Sample preparation and reference materials; ISO: Geneva, Switzerland, 2009.
